# Incorporation of NUPACK-Based Simulation into Classroom and Laboratory Teaching of Nucleic Acids Hybridization for Undergraduate Biochemistry

**DOI:** 10.1021/acs.jchemed.4c01051

**Published:** 2025-06-13

**Authors:** Jinglin Fu, Anthony Monte Carlo, Doris Zheng

**Affiliations:** † Department of Chemistry, 148039 Rutgers University—Camden, Camden, New Jersey 08102, United States; ‡ Center for Computational and Integrative Biology, Rutgers University—Camden, Camden, New Jersey 08102, United States

**Keywords:** Upper-Division Undergraduate, Biochemistry, Laboratory Instruction, Computer-Based Learning, Nucleic Acids/DNA/RNA

## Abstract

The COVID-19 pandemic has accelerated the shift from traditional in-person teaching to remote and online learning, necessitating a more adaptable educational platform to serve the diverse needs of students. Transforming hands-on “wet lab” activities into virtual “dry lab” exercises can promote a more accessible and flexible learning environment, offering innovative methods to improve online teaching outcomes, incorporate interactive components, and provide student support. Here we describe our effort of utilizing NUPACK, a free cloud-based web application, to develop new educational modules on nucleic acids for teaching biochemistry lectures and laboratories. These modules include fundamental topics such as melting temperature, hybridization equilibrium, free energy, secondary folding structures of nucleic acids, and the thermal stability of single-nucleotide polymorphisms. The NUPACK-based DNA computational lab not only provides a hands-on learning experience to enhance students’ understanding of nucleic acid structures, hybridizations, and characteristics but also facilitated the transition to remote learning during the pandemic. Furthermore, these computation-assisted DNA experiments have been extended to engage local high school students at Rutgers University—Camden. This article summarizes the curriculum development and guidelines for the DNA computational lab, aiming to benefit the education of nucleic acids in biochemistry for a wider audience of educators and learners.

## Graphical abstract

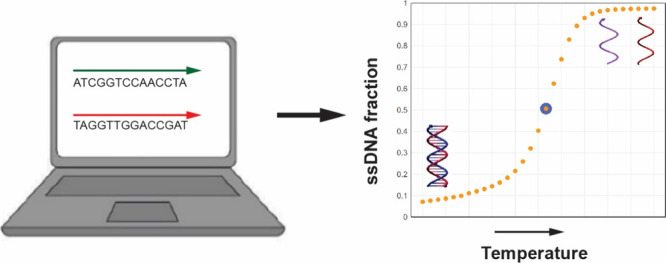

## 1 Introduction

An increasing number of computational tools are being utilized in research and educational activities in chemistry and biochemistry. It is crucial to integrate computational methods into classroom and laboratory teaching of biochemistry.^1,2^ This integration can enhance educational outcomes by bridging the gap between theoretical knowledge and practical applications, offering hands-on experience and enabling complex analyses. Computation-assisted learning is particularly beneficial in scenarios with limited laboratory resources, allowing students to engage in intricate tasks and analyze biochemical molecules and processes. Additionally, the computation-assisted approach makes teaching styles more adaptable to fit diverse student needs. For instance, the COVID-19 pandemic promoted remote and online teaching, requesting a rapid modification and a shift of educational platforms toward remote learning modules. This rapid shift posed a challenge for many subjects and negatively impacted the education outcome of students’ learning, especially for laboratory-based instruction. Converting “wet lab” practices to “dry lab” exercises can facilitate more accessible and flexible remote learning, offering innovative ways to enhance online teaching outcomes, incorporate interactive elements, and support students in this new learning environment.

Nucleic acids are a fundamental and essential topic in biochemistry. DNA and RNA, as key biomolecules, play crucial roles in storing and transmitting genetic information about living organisms. In biochemistry education, exploring the molecular structure, function, and applications of nucleic acids is essential. Some basic elements include single-stranded and double-helix structures of DNA, base pairing rules, secondary structures of single-stranded nucleic acids, thermodynamic properties, and hybridization equilibrium. Practical applications such as polymerase chain reaction and DNA sensory circuits should also be introduced.^3^ Computational tools can enhance the learning outcomes by providing hands-on experience for investigating nucleic acid structures’ functions, properties, and hybridizations.

NUPACK is an evolving online software suite used for simulating or analyzing nucleic acid structures and hybridizations as well as designing complex nucleic acid systems. The software platform was developed by Dr. Niles A. Pierce and his colleagues at the California Institute of Technology,^4,5^ which was supported by the U.S. National Science Foundation and the Molecular Programming Project. NUPACK uses models published by Santa Lucia (1998)^6^ for DNA molecules and Serra and Turner (1995)^7^ and Matthews et al. (1999)^8^ for RNA molecules. It excels in the computation and simulation of the thermodynamic parameters of DNA and RNA strand interactions such as Δ*G* of hybridization, equilibrium, and secondary structures. Widely used by researchers for DNA molecular programming, nucleic acid nanotechnology, and synthetic biology, NUPACK is considered a standard toolbox for analyzing and designing nucleic acid systems.^9,10^ It is open-access and free for individual users with an updated NUPACK Cloud server that accommodates a high volume of users and tasks, making it ideal for educational and research purposes. Additionally, NUPACK can be integrated with advanced programming languages like Python and C for customized applications.^11^

During the COVID-19 pandemic, we developed computational DNA laboratories for remote and online teaching in the biochemistry lectures and laboratories in the Chemistry Department of Rutgers University—Camden. We utilized NUPACK to create new and innovative learning modules on nucleic acids, offering students a visual, hands-on, and interactive experience. This paper summarizes the course development and instructions of the DNA computation lab, which can benefit the education on nucleic acids and biochemistry for a broader audience of teachers and students.

## 2 Methods

NUPACK Cloud web app^12^ is used for the analysis and simulation of nucleic acid structures and hybridizations. Before using NUPACK, teachers and students should register for an individual noncommercial academic subscription, which is temporarily free now. Using the NUPACK Python module and/or source code and modification is also permitted for noncommercial academic purposes only. This allows more user-friendly modification of NUPACK for specific research tasks. The software module and source code should be requested from info@nupack.org, and the redistribution of the software in source form and/or binary form is not permitted.

Other useful tools include online IC_50_ fitting,^13^ Reverse Complement,^14^ and software for Excel or Prism (GraphPad). All data fitting can be completed by the free-access IC_50_ fitting software and Excel if Prism is not available. The detailed instructions for NUPACK are included in the Supporting Information. No unexpected or unusually high safety hazards were encountered.

## 3 Simulation and Analysis of Thermal Denaturation of dsDNA

### 3.1 NUPACK Simulation of Thermal Denaturation of dsDNA

In Figure 1, the thermal melting temperature (*T*_m_) of DNA strands refers to the specific temperature at which 50% of DNA in a sample has transitioned from double-stranded DNA (dsDNA) into single-stranded DNA (ssDNA) due to the heat-induced denaturation of the double-helix structure.^15^ *T*_m_ indicates dsDNA stability, with a higher GC content resulting in a higher *T*_m_ due to increased thermal stability. Additionally, magnesium ions play an important role in stabilizing dsDNA by neutralizing the negative charges on the phosphate backbone. Experimentally, the dissociation of dsDNA into ssDNA can be characterized by the increased absorbance of DNA solutions at 260 nm because of the hyperchromic effect. In dsDNA, the hydrogen bonding between base pairs restricts the resonance in aromatic rings, reducing light absorbance in the helical structure.^16^ An ssDNA solution can absorb 37% more light than a dsDNA solution at the same concentration of total oligonucleotides. Typically, a UV–vis spectrometer is used to measure dsDNA’s thermal denaturation process, with a water or thermal cycler to control the temperature. The quality of the data largely depends on the operational procedure and instrument accuracy. Students may face challenges in obtaining a good thermal-melting curve using a water cycler/UV–vis instrument.

**Figure 1:**
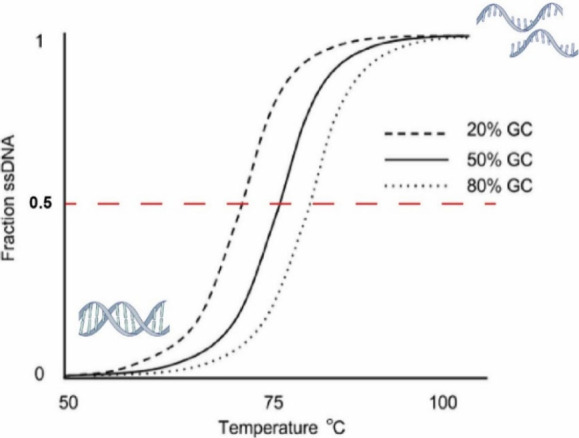
**Thermal denaturation of dsDNA into ssDNA by heating.** *T*_m_ is defined as the temperature at which 50% of DNA has transitioned from dsDNA into ssDNA. *T*_m_ increases with increasing GC content.

NUPACK offers an alternative computation-based “dry lab” to teach students about the melting temperature of DNA hybridization. As shown in Figure 2, NUPACK is used to simulate the thermal denaturation of dsDNA with a given range of temperatures and salt conditions. Students can set up the parameters of “Compute melt” in NUPACK to generate a thermal denaturation graph showing the equilibrium fraction of unpaired ssDNA at different temperatures (Figure 2A,B). The melting temperature can be analyzed by fitting the data with the “Boltzmann Sigmoidal Equation” in GraphPad Prism (Figure 3A),^17^ where *V*_50_ corresponds to the *T*_m_ value. Another method of fitting the melting temperature is to analyze the slope of the thermal denaturation curve by “first-order derivative analysis” in Prism. *T*_m_ can be defined as the temperature at which the maximum absorbance change (d*A*/d*T*) occurs.^12^ Thus, the peak value of the slope analysis approximates *T*_m_ (Figure 3B). While GraphPad Prism is effective for data fitting, the instructor and students may need to purchase licenses. A free online alternative is the “IC_50_/EC_50_” tool, which can calculate the melting temperature by fitting the IC_50_ value of DNA hybridization thermal denaturation (see the Supporting Information).

**Figure 2:**
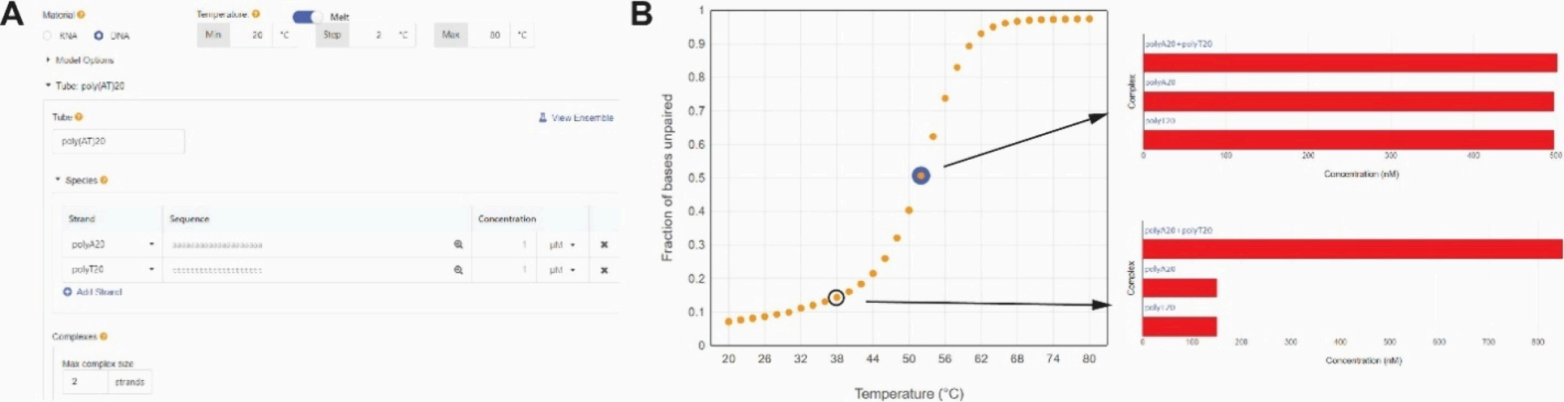
**NUPACK simulation of the thermal denaturation of dsDNA.** (A) Setup for the melting analysis of dsDNA in NUPACK. (B) Calculation of the ssDNA fraction at equilibrium depending on the temperature.

**Figure 3:**
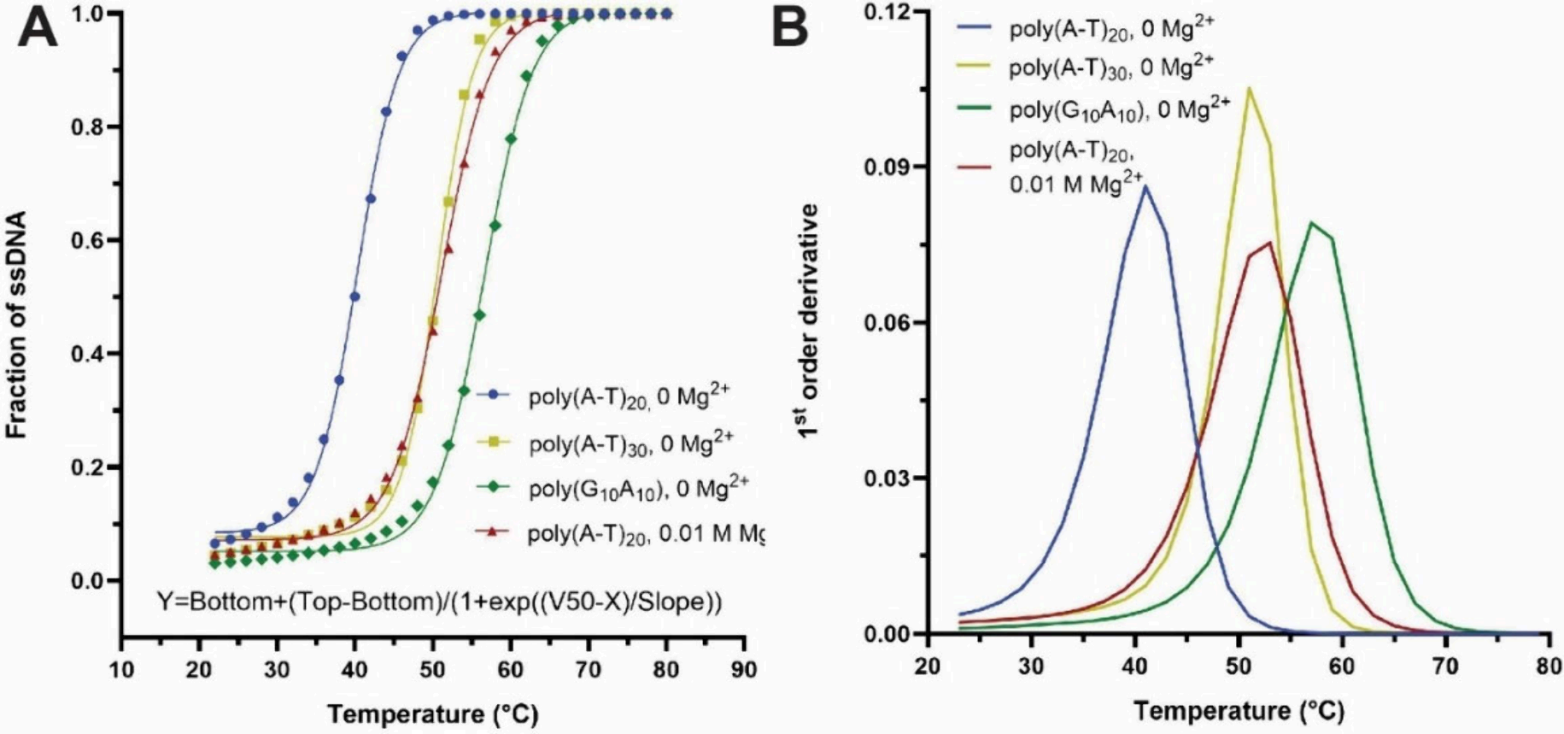
***T*_m_ fitting for thermal denaturation of dsDNA.** (A) *T*_m_ fitting using the Boltzmann sigmoidal model for *V*_50_. (B) *T*_m_ fitting using first-order derivative analysis for the peak temperature.

Using NUPACK and fitting analysis, students investigate the melting temperature of dsDNA hybridization depending on various parameters (Table 1), including the number of base pairs (e.g., poly­(A-T)_20_ vs poly­(A-T)_30_), the variation of GC content (e.g., poly­(A-T)_20_ vs poly­(G_10_A_10_)), and the addition of Mg^2+^. Students should conclude that dsDNA hybridization is more stable with an increased number of base pairs, higher GC content, and the addition of Mg^2+^ to reduce electrostatic repulsion.

**Table 1:** Melting Temperatures for dsDNA Analyzed at Various Magnesium Concentrations, GC Contents, and Lengths

	Poly(A-T)_20_			
Best-Fit Values	0 M Mg^2+^	0.01 M Mg^2+^	Poly(G_10_A_10_)	Poly(A-T)_30_
Bottom	0.092	0.077	0.055	0.080
Top	1.005	1.012	1.016	1.007
*V*_50_ or *T*_m_ (μM)	40.30	51.09	56.54	50.55
*R* ^2^	0.999	0.998	0.998	0.998

### 3.2 Comparison of Melting Temperature Differences for Single-Nucleotide Polymorphism

A single-nucleotide polymorphism (SNP) is a variation found at a specific single nucleotide position in the DNA sequence of the genome among individuals.^18^ For instance, at a specific base position in the human genome, the majority of people may have the nucleotide G, while in a minority, it may be an A. This indicates the presence of an SNP at that specific position, with the two possible nucleotide variations (G or A) being referred to as alleles for that position. While some SNPs do not lead to disorders, certain SNPs are linked to specific diseases and personalized medicine. A mismatch of the base pairing in SNPs can result in a decrease in melting temperature. Polymerase chain reaction (PCR) is a commonly adopted technique for identifying SNPs through the change in *T*_m_ to detect the mismatch hybridization between the target DNA strands and probes.^19^

In the lab, students are asked to analyze a known SNP with ID “rs762551”, which encodes the CYP1A2*1F allele of the CYP1A2 gene.^20^ CYP1A2 is a cytochrome P450 enzyme that is responsible for the metabolism of caffeine and some drugs. An SNP of “CTC­TGT­GGGC **[C/A]** CAGG­ACG­CAT is found within this gene, where a “C” base may appear at the specific base position (red-labeled) in the human genome for some individuals; however, others may show an “A” base instead. This indicates an SNP of the C/A mutation at this specific position. As shown in Table 2, two TaqMan probes are designed to detect the “rs762551” SNP: one probe strand corresponding to the “C” mutation is labeled with a TET fluorophore (tetrachlorofluorescein), and the other probe strand corresponding to the “A” mutation is labeled with a FAM fluorophore (carboxyfluorescein). In the TaqMan PCR assay, the probe strand reports a higher fluorescence when fully matched with the target sequence, while the probe strand with an SNP mismatch reports a weaker fluorescence. The mismatch in DNA hybridization results in a *T*_m_ lower than that of a fully matched hybridization.

**Table 2:**
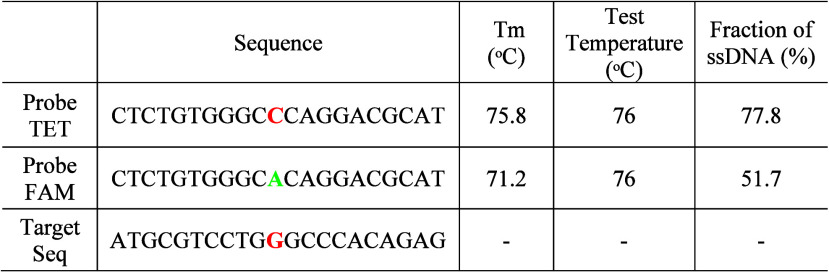
Thermal Melting Analysis of SNPs

To examine the impact of SNP mismatch on the melting temperature, students use NUPACK to simulate the thermal denaturation process of DNA hybridizations between a target and a probe labeled with TET or FAM. By analyzing the thermal denaturation, students should select a test temperature under which the fraction of ssDNA differs the most between TET-Target and FAM-Target.

## 4 Simulation of the Secondary Structure for Nucleic Acid Amplicons

For molecular diagnostic applications, it is necessary to identify an appropriate target sequence. Due to homologies between virulent and benign species, these sequences must be chosen carefully to avoid false positives. This can be an involved bioinformatic process. In nucleic acid diagnosis, viral amplicons are the unique and conserved segments (e.g., E and N genes in SARS-CoV-2) shorter than a few hundred nucleotides. For example, in Figure 4, the E (envelope) and N (nucleocapsid) genes of the SARS-CoV-2 virus are quite conserved across mutations and viral subtypes.^21^ Therefore, amplicons in the E or N gene are used to screen the patient sample and confirm the infection of SARS-CoV-2. The U.S. Centers for Disease Control and Prevention (CDC) published three amplicons from the N gene for identifying the infection of the SARS-CoV-2 virus that caused the COVID-19 pandemic (Table 3).^22^ The sections labeled red are forward primers and antireverse primers for amplification by PCR. The green-labeled sections are the probe sequences for detection.

**Figure 4:**
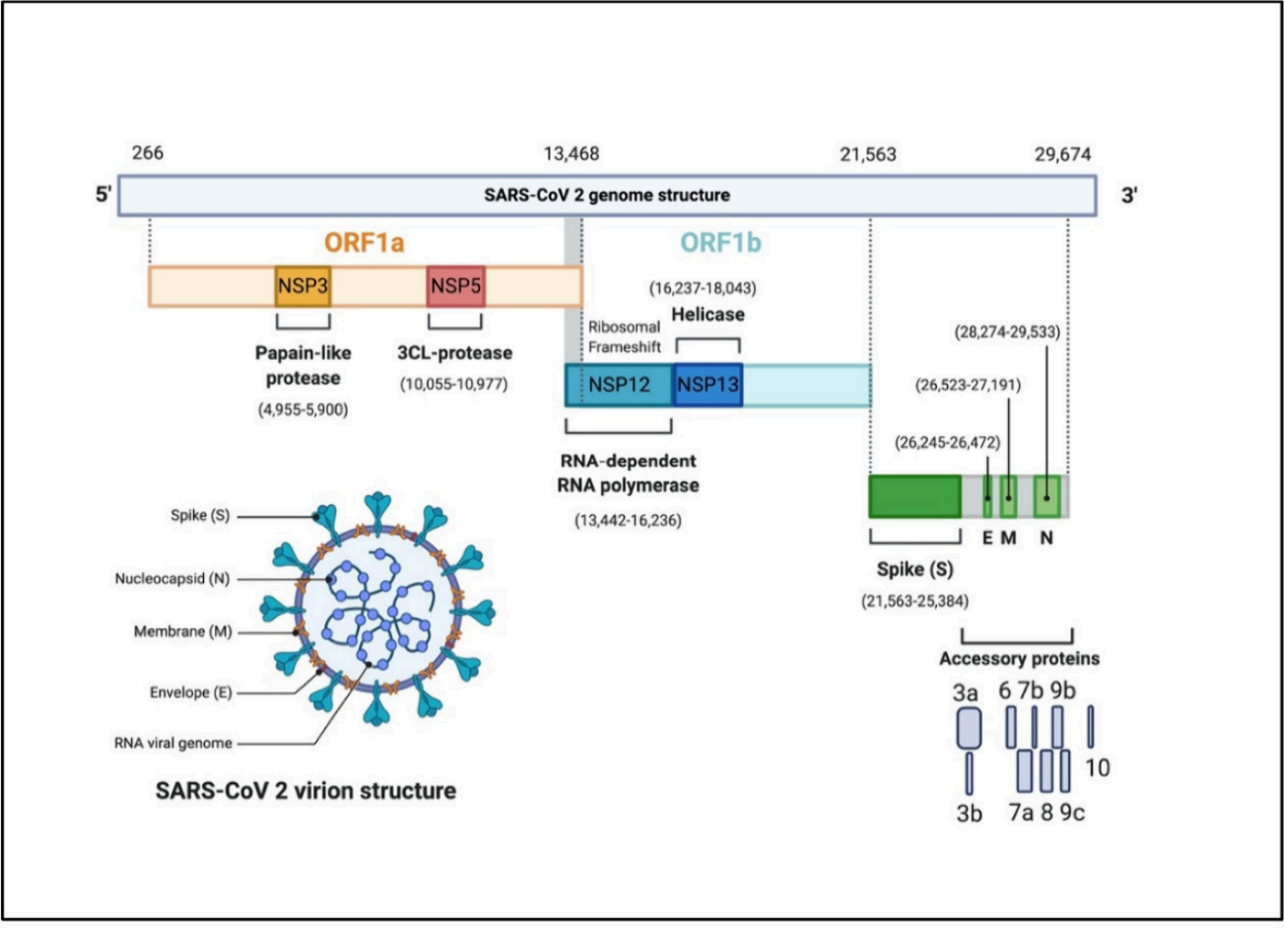
**SARS-CoV-2 genome map.** Diagnosis amplicons use the unique and conserved E and N genes, coding for the viral envelope and nucleocapsid. Reproduced from ref 21. CC BY 4.0.

**Table 3:**
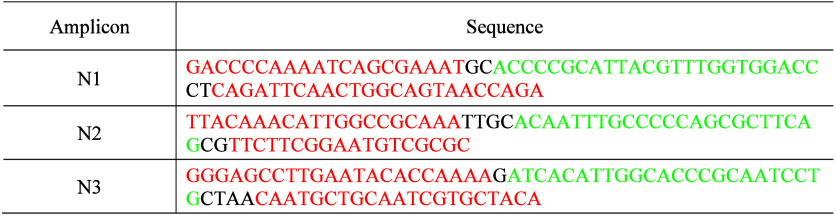
U.S. CDC Published N Gene Amplicons of the SARS-CoV2 Virus for Diagnosing COVID-19 Infection^a^

a The sections labeled in red are forward primers and anti-reverse primers for amplification by PCR. Sections labeled in green are the probe sequences for detection.

In this task, students are instructed to use NUPACK to simulate the secondary structures of DNA amplicons at various temperatures. In Figure 5, at room temperature of 25 °C, three amplicons exhibit stable secondary structures with a large negative Δ*G*. As the temperature increases from 25 °C to 60 and 90 °C, the self-folded secondary structures of the DNA amplicons become less stable, resulting in decreased Δ*G* values. Through simulation, students should understand that the stability of self-folded secondary structures of DNA is highly temperature-dependent.

**Figure 5:**
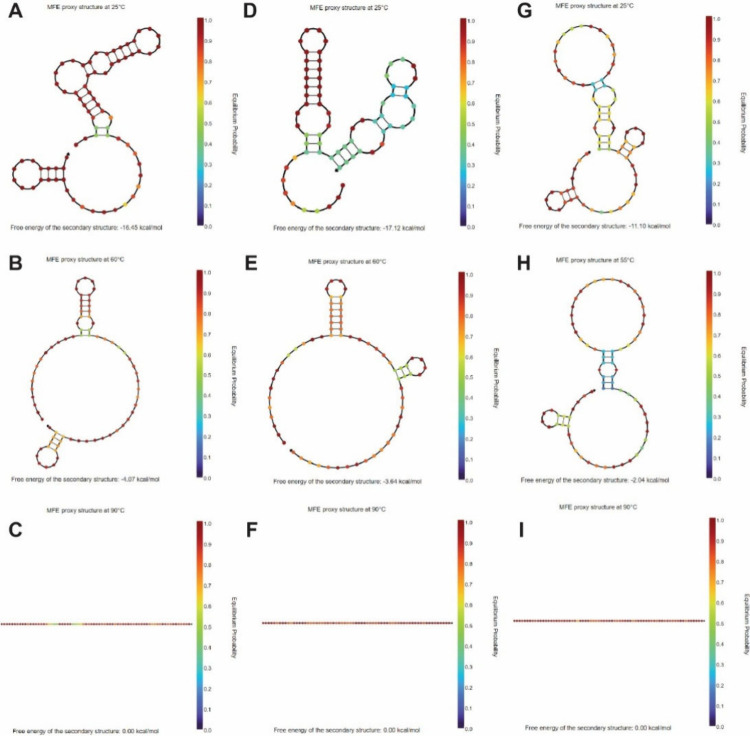
**Simulation of the folding structure and energy for DNA amplicons of SARS-COV2 virus.** (A–C) N1 amplicon at (A) 25 °C, (B) 60 °C, and (C) 90 °C. (D–F) N2 amplicon at (D) 25 °C, (E) 60 °C, and (F) 90 °C. (G–I) N3 amplicon at (G) 25 °C, (H) 55 °C, and (I) 90 °C.

## 5 Classroom Implementation

The DNA computation lab has been implemented into the General Biochemistry II Laboratory at Rutgers University—Camden. Each section of the lab has a maximum capacity of 18 students and usually contains 12–18 students. The lab generally offers two sections for 30–40 students each year. The students are asked to complete a prelab quiz to review the lab and the instructions to prepare for the lab, such as registering for the student account for NUPACK, learning DNA thermal stability, and practicing examples of IC_50_ fitting and first-order derivative analysis.

The DNA computation lab runs in a regular lab schedule of 3 contact hours. The lab starts with instructions for the DNA lab and a step-by-step NUPACK guide. All required instruction information is available in the supporting PPT file. Most students can complete the tasks within a regular lab period. The NUPACK and fitting analyses are quite straightforward to implement. Below are tasks for students to complete in the classroom.

*Task 1. Thermal Denaturation and Melting Temperature Fitting.* All of the NUPACK experiments are set at 0.137 M Na^+^, and the temperature ranges from 20 to 80 °C. The students perform the following:

1.1. NUPACK-generated thermal denaturation graph for poly­(A-T)_20_ and poly­(A-T)_40_.
1.2. NUPACK-generated thermal denaturation graph for poly­(A-T)_20_ and poly­(C-G)_20_.
1.3. NUPACK-generated thermal denaturation graph for poly­(A-T)_20_ at 0 M Mg^2+^ and poly­(A-T)_20_ at 0.01 M Mg^2+^.
1.4. Melting temperature fitting by at least two methods, “Boltzmann Sigmoidal Kinetics” (or “IC_50_”) and “first-order derivative analysis”.
1.5. Summary of the melting temperatures for DNA hybridizations above and drawing a conclusion about what factors affect the melting temperature of dsDNA hybridization.

*Task 2. Thermal Denaturation for Single-Nucleotide Polymorphism.* All of the NUPACK experiments are set at 0.137 M Na^+^. The students perform the following:

2.1. NUPACK-generated thermal denaturation graphs for hybridization of TET with Target and FAM with Target.
2.2. Melting temperature fitting by “IC_50_” and “first-order derivative analysis”.
2.3 Picking a temperature based on the melting temperature fitting with a significant difference in hybridization yield between TET–Target and FAM–Target.
2.4. Drawing a conclusion about how the single-nucleotide mismatch affects the melting temperature of the DNA hybridization.

*Task 3. Simulation of the Secondary Folding Structures of ssDNA Amplicons.* The students perform the following:

3.1. Simulation of the secondary folding structures of N1, N2, and N3 ssDNA amplicons at 25 °C and finding Δ*G* of the structures.
3.2. Simulation of the secondary folding structures of N1, N2, and N3 ssDNA amplicons at 60 °C and finding Δ*G* of the structures.
3.3. Simulation of the secondary folding structures of N1, N2, and N3 ssDNA amplicons at 90 °C and finding Δ*G* of the structures.
3.4. Drawing a conclusion about how the secondary folding structures of ssDNA change depending on the temperature and their structural Δ*G*.

## 6 Student Participation and Feedback

Since the COVID-19 pandemic, the DNA simulation lab has been incorporated into the General Biochemistry Lab, which hundreds of students have taken in the past 10 years at Rutgers University—Camden. Besides, this DNA simulation lab has also been introduced to high school students who were sponsored by the High School Internships Program (HSIP) from the Army Education Outreach Programs (AEOP). HSIP has sponsored two or three local high school students per year for 8–10 week paid summer research experiences at Rutgers University—Camden since 2015. Students can apply directly to HSIP from the AEOP website and are interviewed and selected by the research mentor. The research rotation students in the Fu lab are required to take this DNA simulation training. With the completion of DNA simulation training, students can participate in more complex computation-aided designs and experiments. To enhance K–12 education, we also invited teachers from local high schools to participate in the DNA simulation training. Table 4 summarizes the student training and participation in the NUPACK-based DNA simulation lab in the past 5 years.

**Table 4:** Students’ Participation in the DNA Simulation Lab

Biochem II Lab	Summer Research Apprenticeship	New Students Training for Research
2020–present	2020–present	2020–present
∼30–40 undergraduates per year; 222 total	2–3 high school students per summer; 10 total	3–4 rotation students per year; 16 total

We used the “DNA melting temperature” experiment to evaluate the students’ learning outcomes, which was included in both the wet lab and the dry lab. In this lab, the primary learning goal includes (1) understanding the DNA thermal melting process, (2) fitting the melting temperature for DNA hybridization, and (3) investigating the factors that affect the melting temperature of DNA hybridization. Therefore, the laboratory relies on high-quality data and fitting analysis. While the wet lab can offer students real hands-on experience in sample preparation and experiment operation, it is not the primary goal of the “DNA melting temperature” lab. In the wet lab, students often had trouble obtaining high-quality data for fitting due to equipment limitations and incorrect procedures. For example, the traditional water-bath UV spectrometer may not produce good results due to poor temperature control and slow heating and cooling steps. The students may also fail the experiment due to mistakes in sample preparation and uncareful operation. Additionally, due to the slow data collection, students can investigate only limited parameters and conditions for DNA hybridizations. To improve the data quality and throughput, we asked students to use the NUPACK-based simulation to generate various data sets of the DNA thermal melting process and adjust the input parameters and conditions. These simulated data sets are used to perform fitting algorithms to calculate the melting temperature.

The student’s performances in the wet and dry labs for the DNA melting temperature are compared in Figure 6. There are more failed or incomplete lab reports for the wet lab (4) than for the dry lab (2). The average score of a lab report improved from 82.5 for the wet lab to 94.5 for the dry lab. As explained above, in the wet lab, the instructor and students may struggle to acquire a good data set for DNA thermal melting, which could be affected by the instrument setup, sample preparation, and operation. The NUPACK-based simulation can substantially improve the students’ understanding of DNA melting temperature by providing a hands-on and interactive approach to learning. Using this, students can visualize and analyze the stability of DNA duplexes when they manipulate various parameters, such as sequence composition, length, and ion concentration. Such a simulation-based learning approach is particularly useful for a lab focusing more on data analysis and parameter investigation rather than the wet lab experience. General Biochemistry Lab I at Rutgers University—Camden includes mixed topics of wet and dry laboratories, providing students with experience in basic laboratory skills, simulation, and data analysis.

**Figure 6:**
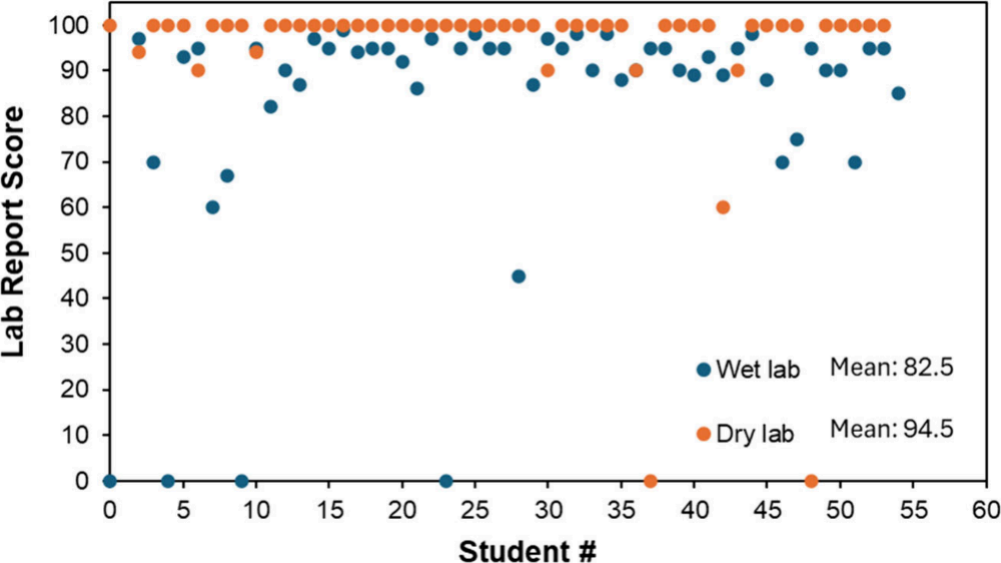
**Assessment of student learning outcomes for DNA melting temperature with the wet lab (blue dots) and the NUPACK-based dry lab (orange dots).** The number of incomplete lab reports was reduced from four for the wet lab to two for the dry lab. The average score of a lab report improved from 82.5 for the wet lab to 94.5 for the dry lab.

## 7 Conclusion

In summary, we have demonstrated the use of NUPACK in teaching computation-based DNA laboratories for undergraduate students in General Biochemistry. NUPACK is a user-friendly and free online tool for predicting DNA hybridization equilibrium, simulating DNA/RNA structures, and calculating the hybridization energy. By integrating NUPACK into the learning process, students can explore factors affecting DNA hybridization’s melting temperature and stability, such as magnesium concentration, GC content, and length. Additionally, students practice analyzing melting temperature differences in SNPs, commonly used in PCR. They also learn about data-fitting tools for analyzing the thermal denaturation process, including fitting models such as IC_50_, Boltzmann sigmoidal kinetics, and first-order derivative analysis. Apart from hybridization studies, students are exposed to simulating the secondary folding structures of ssDNA amplicons from the SARS-CoV-2 viral genome. This hands-on experience of basic DNA computation provides a unique learning opportunity for students in the Biochemistry class, enhancing their understanding of nucleic acid structures, hybridizations, and properties. Students interested in further exploration can use NUPACK to design and simulate complex DNA hybridization systems such as toehold-mediated strand displacement and sandwich hybridization and participate in undergraduate research on DNA nanosystems. This computational-based approach to DNA learning has also been introduced to summer research apprenticeship programs for high school students (e.g., High School Apprenticeships by the Army Education Outreach Program).

## Supplementary Material



## Notes

Lab instruction material and NUPACK step-by-step instructions (PDF)

## References

[1] McDonald A. R., Roberts R., Koeppe J. R., Hall B. L. (2022). Undergraduate structural
biology education: A shift from users to developers of computation
and simulation tools. Curr. Opin. Struct. Biol..

[2] Sung R. J., Wilson A. T., Lo S. M., Crowl L. M., Nardi J., St. Clair K., Liu J. M. (2020). BiochemAR: An Augmented Reality Educational
Tool for Teaching Macromolecular Structure and Function. J. Chem. Educ..

[3] Santiago-McRae E., Oh S. W., Carlo A. M., Bar O., Guan E., Zheng D., Grgicak C., Fu J. (2022). Rapid Nucleic
Acid
Reaction Circuits for Point-of-care Diagnosis of Diseases. Curr. Top. Med. Chem..

[4] Dirks R. M., Pierce N. A. (2003). A partition function
algorithm for nucleic acid secondary
structure including pseudoknots. J. Comput.
Chem..

[5] Zadeh J. N., Steenberg C. D., Bois J. S., Wolfe B. R., Pierce M. B., Khan A. R., Dirks R. M., Pierce N. A. (2011). NUPACK: Analysis
and design of nucleic acid systems. J. Comput.
Chem..

[6] Santa
Lucia J. C. (1998). A unified view of polymer, dumbbell,
and oligonucleotide DNA nearest-neighbor thermodynamics. Proc. Natl. Acad. Sci. U. S. A..

[7] Serra M. J., Turner D. H. (1995). Predicting thermodynamic
properties of RNA. Methods Enzymol..

[8] Mathews D. H., Sabina J., Zuker M., Turner D. H. (1999). Expanded sequence
dependence of thermodynamic parameters improves prediction of RNA
secondary structure. J. Mol. Biol..

[9] Fornace M. E., Huang J., Newman C. T., Porubsky N. J., Pierce M. B., Pierce N. A. (2022). NUPACK: Analysis
and Design of Nucleic Acid Structures,
Devices, and Systems. ChemRxiv.

[10] Bellaousov S., Kayedkhordeh M., Peterson R. J., Mathews D. H. (2018). Accelerated RNA
secondary structure design using preselected sequences for helices
and loops. RNA.

[11] Hong F., Ma D., Wu K., Mina L. A., Luiten R. C., Liu Y., Yan H., Green A. A. (2020). Precise and Programmable Detection of Mutations Using
Ultraspecific Riboregulators. Cell.

[12] NUPACK Cloud. https://www.nupack.org/overview.

[13] IC50 Calculator. https://www.aatbio.com/tools/ic50-calculator.

[14] Reverse Complement. https://www.bioinformatics.org/sms/rev_comp.html.

[15] Mergny J. L., Lacroix L. (2003). Analysis of thermal
melting curves. Oligonucleotides.

[16] Ackerman M. M., Ricciardi C., Weiss D., Chant A., Kraemer-Chant C. M. (2016). Analyzing
Exonuclease-Induced Hyperchromicity by UV Spectroscopy: An Undergraduate
Biochemistry Laboratory Experiment. J. Chem.
Educ..

[17] Wienken C. J., Baaske P., Duhr S., Braun D. (2011). Thermophoretic melting
curves quantify the conformation and stability of RNA and DNA. Nucleic Acids Res..

[18] Sherry S. T., Ward M., Sirotkin K. (1999). dbSNP—database for single
nucleotide polymorphisms and other classes of minor genetic variation. Genome Res..

[19] Broccanello C., Chiodi C., Funk A., McGrath J. M., Panella L., Stevanato P. (2018). Comparison of three PCR-based assays for SNP genotyping
in plants. Plant Methods.

[20] Wang H., Zhang Z., Han S., Lu Y., Feng F., Yuan J. (2012). CYP1A2 rs762551 polymorphism contributes
to cancer susceptibility:
a meta-analysis from 19 case-control studies. BMC Cancer.

[21] Alanagreh L., Alzoughool F., Atoum M. (2020). The Human Coronavirus Disease COVID-19:
Its Origin, Characteristics, and Insights into Potential Drugs and
Its Mechanisms. Pathogens.

[22] Lu X. (2020). US CDC Real-Time Reverse
Transcription PCR Panel for Detection of
Severe Acute Respiratory Syndrome Coronavirus 2. Emerging Infect. Dis..

