# Supplementary material for: Incorporation of NUPACK-Based Simulation into Classroom and Laboratory Teaching of Nucleic Acids Hybridization for Undergraduate Biochemistry
Source: J Chem Educ. 2025 Jun 13;102(7):3010–7. doi: 10.1021/acs.jchemed.4c01051 (PMC12333017; doi:10.1021/acs.jchemed.4c01051)
Supplement: Supplementary file 1 [file ed4c01051_si_001.pdf]

Supporting Information

Incorporation of NUPACK-based Simulation into Classroom and Laboratory Teaching of Nucleic Acids Hybridization for Undergraduate Biochemistry

Jinglin Fu,[1, 2] Anthony Monte Carlo[1] and Doris Zheng[1]

[1]Department of Chemistry, Rutgers University–Camden, Camden, NJ 08102, USA.

[2]Center for Computational and Integrative Biology, Rutgers University–Camden, Camden, NJ 08102, USA.

[*]Corresponding Author: Jinglin Fu, jf604@camden.rutgers.edu

Table of Contents

Lab Instruction	S3- S13
Pre-Lab Quiz	S14
Step by Step NUPACK Instruction	S15-S39

Lab: Simulation of DNA hybridizations by NUPACK

I. Introduction

An increasing number of computational tools are being utilized in research and educational activities in Chemistry and Biochemistry. It is crucial to integrate computational methods into classroom and laboratory teaching of Biochemistry.[1, 2] This integration can enhance educational outcomes by bridging the gap between theoretical knowledge and practical applications, offering hands-on experience, and enabling complex analyses. Computation-assisted learning is particularly beneficial in scenarios with limited laboratory resources, allowing students to engage in intricate tasks and analyze biochemical molecules and processes. Additionally, the computation-assisted approach makes teaching styles more adaptable to fit diverse student needs. For instance, the COVID-19 pandemic has promoted remote and online teaching, requesting a rapid modification and shift of educational platforms toward remote learning modules. This rapid shift posed a challenge for many subjects and negatively impacted the education outcome of students learning, especially for laboratory-based instruction. Converting "wet lab" practices to "dry lab" exercises can facilitate more accessible and flexible remote learning, offering innovative ways to enhance online teaching outcomes, incorporate interactive elements, and support students in this new learning environment.

DNA and RNA, as key biomolecules, play crucial roles in storing and transmitting genetic information in living organisms. In Biochemistry education, exploring the molecular structure, function, and applications of nucleic acids is essential. Some basic elements include single-stranded and double-helix structures of DNA, base pairing rules, secondary structures of single-stranded nucleic acids, thermodynamic properties, and hybridization equilibrium. Practical applications such as polymerase chain reaction and DNA sensory circuits should also be introduced.[3] Computational tools can enhance the learning outcome by providing hands-on experience for investigating nucleic acid structures' functions, properties, and hybridizations.

NUPACK is an evolving online software suite used for simulating or analyzing nucleic acid structures and hybridizations, as well as designing complex nucleic acid systems. The software platform was developed by Dr. Niles A. Pierce and his colleagues at the California Institute of Technology, [4, 5] which was supported by the US National Science Foundation and the

Molecular Programming Project. NUPACK uses models published by Santa Lucia (1998)[6] for DNA molecules, Serra and Turner (1995)[7], and Matthews et al. (1999)[8] for RNA molecules. It excels in the computation and simulation of the thermodynamic parameters of DNA and RNA strand interactions, such as the ΔG of hybridizations, equilibrium, and secondary structures. Widely used by researchers for DNA molecular programming, nucleic acid nanotechnology, and synthetic biology, NUPACK is considered a standard toolbox for analyzing and designing nucleic acid systems.[9, 10] It is open-access and free for individual users, with an updated NUPACK Cloud server that accommodates a high volume of users and tasks, making it ideal for educational and research purposes. Additionally, NUPACK can be integrated with advanced programming languages like Python and C for customized applications.[11]

In this lab, we will use NUPACK to investigate fundamental properties of nucleic acids, including melting temperature, hybridization equilibrium, free energy, secondary folding structures of nucleic acids, and the thermal stability of single-nucleotide polymorphisms.

II. Lab Goals

a. Learn how to use NUPACK for the basic DNA/RNA calculation and simulation.

b. NUPACK-based Simulation of Thermal Denaturation and Melting Temperature Fitting.

c. Thermal Denaturation for Single Nucleotide Polymorphism.

d. Simulation of the secondary folding structures of ssDNA amplicons.

III. Methods and Resources

NUPACK Cloud Web APP (https://www.nupack.org/overview) is used for the analysis and simulation of nucleic acid structures and hybridizations. Before using NUPACK, teachers and students should register for a free account for a non-commercial academic subscription (currently free). Using the NUPACK Python module and/or source code and modification is also permitted for non-commercial academic purposes only. This allows more user-friendly modification of NUPACK for specific research tasks. The software module and source code should be requested

from info@nupack.org, and redistributing the Software in source form and/or binary form is not permitted.

Other useful tools include online IC50 fitting (https://www.aatbio.com/tools/ic50-calculator), reverse complement (https://www.bioinformatics.org/sms/rev_comp.html), as well as software for Excel and Prism (GraphPad). Step by Step Instructional PowerPoints for NUPACK.

IV. Lab Tasks.

IV.1 NUPACK Simulation of The Thermal Denaturation for dsDNA

In **Figure S1**, the thermal melting temperature (Tm) of DNA strands refers to the specific temperature at which 50% of DNA in a sample transitions from double-stranded DNA (dsDNA) into single-stranded DNA (ssDNA) due to the heat-induced denaturation of the double helix structure.[12] T_m indicates dsDNA stability, with higher GC content resulting in a higher T_m due to increased thermal stability. Additionally, magnesium ions play an important role in stabilizing dsDNA by neutralizing the negative charges on the phosphate backbone. Experimentally, the dissociation of dsDNA into ssDNA can be characterized by the increased absorbance of DNA solutions at 260 nm because of the hyperchromic effect. In dsDNA, the hydrogen bonding between base pairs restricts the resonance in aromatic rings, reducing light absorbance in the helical structure.[13] ssDNA solution can absorb 37% more light than dsDNA solution at the same concentration of total oligonucleotides. Typically, a UV-VIS spectrometer is used to measure dsDNA's thermal denaturation process, with a water or thermal cycler to control temperature.

NUPACK offers an alternative computation-based “dry lab” to teach students about the melting temperature of DNA hybridization. As shown in **Figure S2**, NUPACK is used to simulate the thermal denaturation of dsDNA with a given range of temperature and salt conditions. Students can set up the parameters of “Compute melt” in NUPACK to generate a thermal denaturation graph showing the equilibrium fraction of unpaired ssDNA at different temperatures (**Figure S2A and B**). For detailed procedures, please read the instructional PPT for NUPACK. The melting temperature can be analyzed by fitting the data with the “Boltzmann Sigmoidal Equation” in GraphPad Prism (**Figure S3A**),[14], which V50 corresponds to the Tm value. Another method of fitting the melting temperature is to analyze the slope of the thermal denaturation curve by "the

first-order derivative analysis" in Prism. Tm can be defined as the temperature at which the maximum absorbance change (dA/dT) occurs.[12] Thus, the peak value of the slope analysis approximates the T_m (**Figure S3B**). While GraphPad Prism is effective for data fitting, the instructor and students may need to purchase licenses. A free online alternative is the "IC50/EC50" tool, which can calculate the melting temperature by fitting the IC50 value of DNA hybridization thermal denaturation (see the instructional PPT).

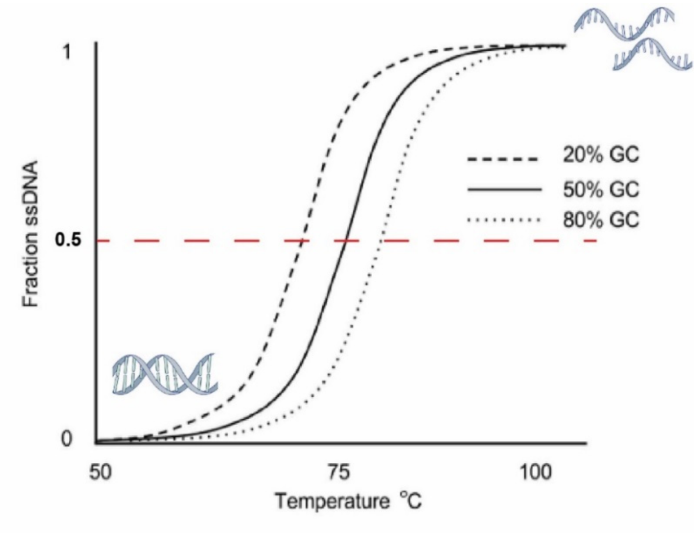

Figure S1. The thermal denaturation of dsDNA into ssDNA by heating. The definition of T_m is at which temperature 50% of DNA transitions from dsDNA into ssDNA. T_m increases with a higher GC component.

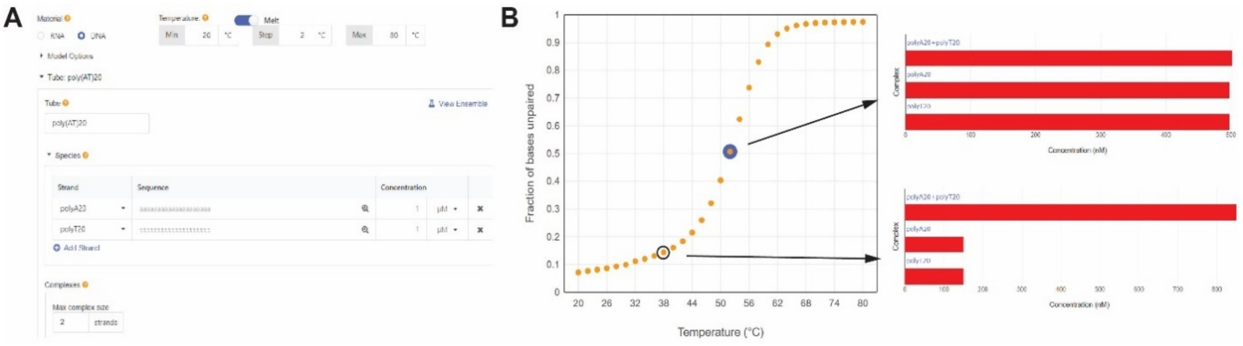

Figure S2. NUPACK simulation of the thermal denaturation of dsDNA. (A) Set up the melting analysis of dsDNA in NUPACK; (B) Calculate the ssDNA fraction in equilibrium depending on the temperature.

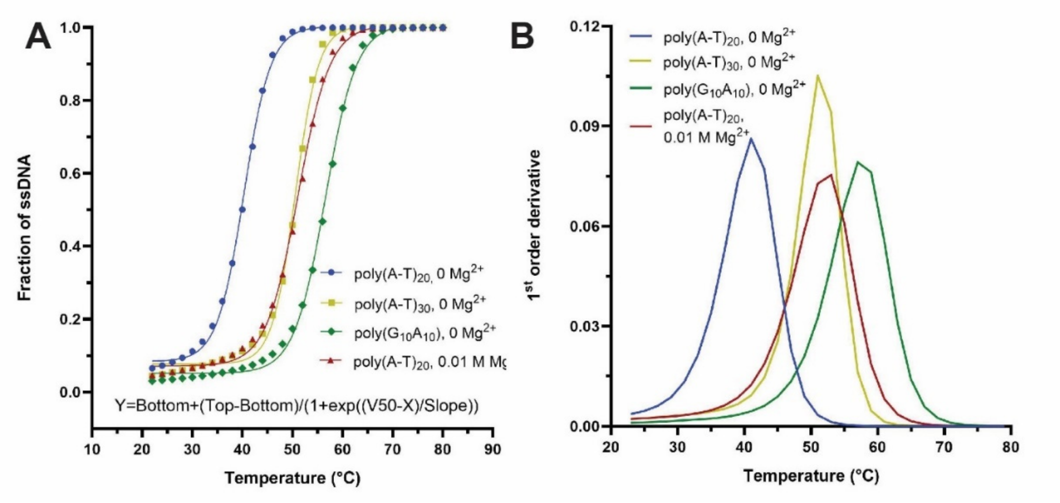

Figure S3. T_m Fitting for thermal denaturation of dsDNA by (A) Boltzmann sigmoidal model for V50 and **(B)** the 1st order derivative analysis for peak temperature.

Table S1. The melting temperature for dsDNA was analyzed at various magnesium concentrations, GC content, and length.

Best-fit values	poly(A-T)$_{20}$, 0 Mg^{2+}	poly(A-T)$_{20}$, 0.01 M Mg^{2+}	poly($G_{10}A_{10}$)	poly(A-T)$_{30}$
Bottom	0.092	0.077	0.055	0.080
Top	1.005	1.012	1.016	1.007
V50 or Tm (μM)	40.30	51.09	56.54	50.55
R squared	0.999	0.998	0.998	0.998

Using NUPACK and fitting analysis, it can investigate the melting temperature of dsDNA hybridization depending on various parameters (**Table S1**), including the number of base pairs

(e.g., poly(A-T)$_{20}$ *vs.* poly(A-T)$_{30}$), the variation of GC% (e.g., poly(A-T)$_{20}$ *vs.* poly($G_{10}A_{10}$)) and the addition of Mg^{2+}. Students will conclude that dsDNA hybridization will be more stable for the increased number of base pairs, the higher GC%, and adding Mg^{2+} to reduce electrostatic repulsion.

Task 1. Thermal Denaturation and Melting Temperature Fitting

All NUPACK experiments are set at 0.137 M Na^+, temperature ranges from 20 ^oC to 80 ^oC.

1.1. NUPACK-generated thermal denaturation graph for poly(A-T)$_{20}$ and poly (A-T)$_{40}$
1.2. NUPACK-generated thermal denaturation graph for poly(A-T)$_{20}$ and poly (C-G)$_{20}$
1.3. NUPACK-generated thermal denaturation graph for poly(A-T)$_{20}$ at 0 Mg^{2+} and poly(A-T)$_{20}$ at 0.01 M Mg^{2+}
1.4. Melting temperature fitting by at least two methods of "Boltzmann Sigmoidal Kinetics" (or IC50) and "the first order derivative analysis."
1.5. Summary of the melting temperatures for DNA hybridizations above. Can you conclude what factors affect the melting temperature of dsDNA hybridization?

IV.2. Compare Melting Temperature Difference for Single-Nucleotide Polymorphism

A single nucleotide polymorphism (SNP) is a variation found at a specific single nucleotide position in the DNA sequence of the genome among individuals.[15] For instance, at a specific base position in the human genome (**Figure S4**), most people may have the nucleotide G, while in a minority, it may be an A. This indicates the presence of an SNP at that specific position, with the two possible nucleotide variations - G or A - being referred to as alleles for that position. While some SNPs do not lead to disorders, certain SNPs are linked to specific diseases and personalized medicine. A mismatch of the base pairing in SNPs can result in a decreased melting temperature. Polymerase chain reaction (PCR) is a commonly adopted technique for identifying SNPs through the change in T_m to detect the mismatch hybridization between the target DNA strands and probes.[16]

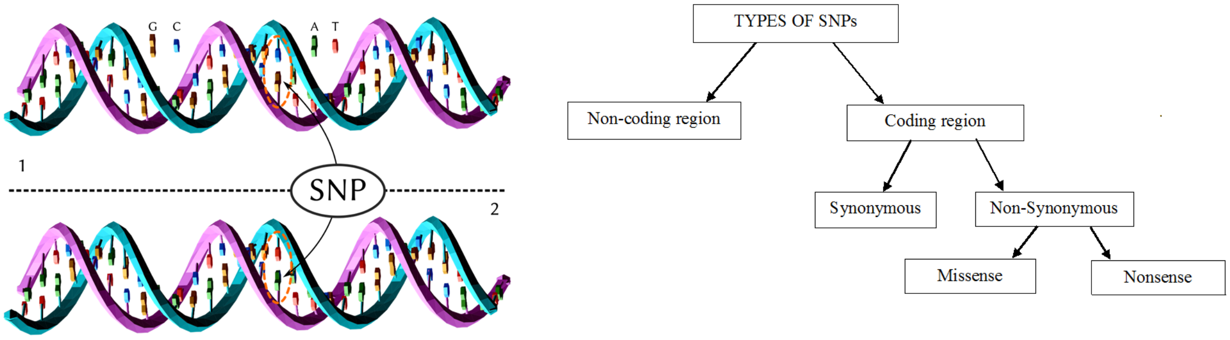

Figure S4. A single nucleotide polymorphism (SNP) and its subtypes. https://en.wikipedia.org/wiki/Single-nucleotide_polymorphism#/media/File:Dna-SNP.svg

In the lab, we will analyze a known SNP ID of "rs762551", which encodes the CYP1A2*1F allele of the CYP1A2 gene.[17] CYP1A2 is a cytochrome P450 enzyme responsible for the metabolism of caffeine and some drugs. An SNP of "CTCTGTGGGC**[C/A]** CAGGACGCAT is found within this gene, where a "C" base may appear at the specific base position (red labeled) in the human genome for some individuals; however, others may show an "A" base instead. This indicates an SNP of C/A mutation at this specific position. As shown in **Table S2**, two TaqMan probes are designed to detect the "rs762551" SNP: one probe strand corresponding to "C" mutation is labeled with a TET fluorophore (tetrachlorofulorescein), and the other probe strand corresponding to "A" mutation is labeled with a FAM fluorophore (carboxyfluorescein). In the TaqMan PCR assay, the probe strand will report a higher fluorescence when fully matched with the target sequence, while the probe strand with an SNP mismatch reports a weaker fluorescence. The mismatch in DNA hybridization results in a lower T_m than a fully matched hybridization.

To examine the impact of SNP mismatch on melting temperature, students will use NUPACK to simulate the thermal denaturation process of DNA hybridizations between a target and a probe labeled with TET or FAM. By analyzing the thermal denaturation, students will select a test temperature under which the fraction of ssDNA differs the most between TET-Target and FAM-Target.

Table S2. The thermal melting analysis of SNPs.

	Sequence	Tm (°C)	Test Temperature (°C)	Fraction of ssDNA (%)
Probe TET	CTCTGTGGGCCCAGGACGCAT	?	76	?
Probe FAM	CTCTGTGGGCACAGGACGCAT	?	76	?
Target Seq	ATGCGTCCTGGGCCCACAGAG	-	-	-

Task 2. Thermal Denaturation for Single Nucleotide Polymorphism

All NUPACK experiments are set at 0.137 M Na^+

2.1. NUPACK-generated thermal denaturation graphs for TET hybridizing with Target and FAM hybridizing with Target.

2.2. Melting temperature fitting by "IC50" and "the first order derivative analysis."

2.3. Pick a temperature based on the melting temperature fitting with a significant difference in hybridization yield between TET-Target and FAM-Target.

2.4. Can you conclude how the single-nucleotide mismatch affects the melting temperature of the DNA hybridization?

IV.3. Simulation of the secondary structure for nucleic acids amplicons.

For molecular diagnostic applications, it is necessary to identify an appropriate target sequence. Due to homologies between virulent and benign species, these sequences must be chosen carefully to avoid false positives. This can be an involved bioinformatic process. In nucleic acid diagnosis, viral amplicons are the unique and conservative segments (e.g., E and N gene in SARS-COV-2) shorter than a few hundred nucleotides. For example, in **Figure S5**, the E (envelop) and N (nucleocapsid) genes of the SARS-COV-2 virus are quite conservative across mutations and viral subtypes.[18] Thereby, amplicons in the E or N gene are used to screen the patient sample and to confirm the infection of SARS-COV-2. The United States Center for Diseases and Control

(CDC) has published three amplicons from the N gene to identify the infection of the SARS-COV-2 virus that caused the COVID-19 pandemic (**Table S3**).[19] The sections labeled red are forward primers and anti-reverse primers for amplification by PCR. Green is the probe sequence for detection.

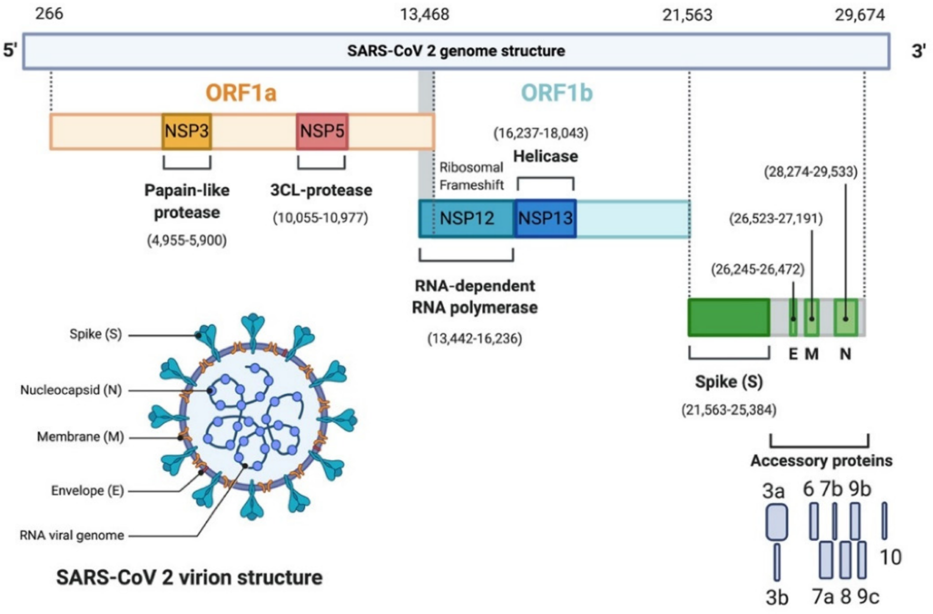

Figure S5. SARS COV-2 genome map. Diagnosis amplicons use the unique and conservative gene at E and N gene, coating for the viral envelope and nucleocapsid. The figure is reproduced with permission under the Creative Commons Attribution (CC BY) license from *Pathogens*.[18]

In this task, we use NUPACK to simulate secondary structures of DNA amplicons at various temperatures. As the temperature increases from 25 °C to 60 °C and 90 °C, the self-folded secondary structures of the DNA amplicons become less stable, resulting in lower ΔG values. Through simulation, students will understand that the stability of self-folded secondary structures of DNA is highly temperature-dependent.

Table S3. US CDC published N gene amplicons of SARS-COV2 virus for diagnosing COVID-19 infection. The sections labeled red are forward primers and anti-reverse primers for amplification by PCR. Green is the probe sequence for detection.

Amplicon	Sequence
N1	GACCCCAAAATCAGCGAAATGCACCCCGCATTACGTTTGGTGGACC CTCAGATTCAACTGGCAGTAACCAGA
N2	TTACAAACATTGGCCGCAAATTGCACAATTTGCCCCCAGCGCTTCA GCGTTCTTCGGAATGTCGCGC
N3	GGGAGCCTTGAATACACCAAAAGATCACATTGGCACCCGCAATCCT GCTAACAATGCTGCAATCGTGCTACA

Task 3. Simulation of the secondary folding structures of ssDNA amplicons

3.1. Simulation of the secondary folding structures of N1, N2, and N3 ssDNA amplicons at 25 ^{o}C and ΔG of structures.

3.2. Simulation of the secondary folding structures of N1, N2, and N3 ssDNA amplicons at 60 ^{o}C and ΔG of structures.

3.3. Simulation of the secondary folding structures of N1, N2, and N3 ssDNA amplicons at 90 ^{o}C and ΔG of structures.

3.4. Can you conclude how the secondary folding structures of ssDNA change depending on the temperature and their structural ΔG?

V. Data Report

A date report is required to complete all questions from Task 1 – Task 3.

Reference

1. McDonald, A.R., Roberts, R., Koeppe, J.R. & Hall, B.L. Undergraduate structural biology education: A shift from users to developers of computation and simulation tools. *Current Opinion in Structural Biology* **72**, 39-45 (2022).

2. Sung, R.-J. et al. BiochemAR: An Augmented Reality Educational Tool for Teaching Macromolecular Structure and Function. *Journal of Chemical Education* **97**, 147-153 (2020).
3. Santiago-McRae, E. et al. Rapid Nucleic Acid Reaction Circuits for Point-of-care Diagnosis of Diseases. *Current topics in medicinal chemistry* **22**, 686-698 (2022).
4. Dirks, R.M. & Pierce, N.A. A partition function algorithm for nucleic acid secondary structure including pseudoknots. *J Comput Chem* **24**, 1664-1677 (2003).
5. Zadeh, J.N. et al. NUPACK: Analysis and design of nucleic acid systems. *Journal of Computational Chemistry* **32**, 170-173 (2011).
6. SantaLucia, J., Jr. A unified view of polymer, dumbbell, and oligonucleotide DNA nearest-neighbor thermodynamics. *Proc Natl Acad Sci U S A* **95**, 1460-1465 (1998).
7. Serra, M.J. & Turner, D.H. in Methods in Enzymology, Vol. 259. (ed. A. Press) 242-261 (1995).
8. Mathews, D.H., Sabina, J., Zuker, M. & Turner, D.H. Expanded sequence dependence of thermodynamic parameters improves prediction of RNA secondary structure. *J Mol Biol* **288**, 911-940 (1999).
9. Fornace ME, H.J., Newman CT, Porubsky NJ, Pierce MB, Pierce NA. NUPACK: Analysis and Design of Nucleic Acid Structures, Devices, and Systems *ChemRxiv.* (2022).
10. Bellaousov, S., Kayedkhordeh, M., Peterson, R.J. & Mathews, D.H. Accelerated RNA secondary structure design using preselected sequences for helices and loops. *Rna* **24**, 1555-1567 (2018).
11. Hong, F. et al. Precise and Programmable Detection of Mutations Using Ultraspecific Riboregulators. *Cell* **180**, 1018-1032.e1016 (2020).
12. Mergny, J.L. & Lacroix, L. Analysis of thermal melting curves. *Oligonucleotides* **13**, 515-537 (2003).
13. Ackerman, M.M., Ricciardi, C., Weiss, D., Chant, A. & Kraemer-Chant, C.M. Analyzing Exonuclease-Induced Hyperchromicity by UV Spectroscopy: An Undergraduate Biochemistry Laboratory Experiment. *Journal of Chemical Education* **93**, 2089-2095 (2016).
14. Wienken, C.J., Baaske, P., Duhr, S. & Braun, D. Thermophoretic melting curves quantify the conformation and stability of RNA and DNA. *Nucleic Acids Res* **39**, e52 (2011).
15. Sherry, S.T., Ward, M. & Sirotkin, K. dbSNP—database for single nucleotide polymorphisms and other classes of minor genetic variation. *Genome research* **9**, 677-679 (1999).
16. Broccanello, C. et al. Comparison of three PCR-based assays for SNP genotyping in plants. *Plant Methods* **14**, 28 (2018).
17. Wang, H. et al. CYP1A2 rs762551 polymorphism contributes to cancer susceptibility: a meta-analysis from 19 case-control studies. *BMC Cancer* **12**, 528 (2012).
18. Alanagreh, L.a., Alzoughool, F. & Atoum, M. The Human Coronavirus Disease COVID-19: Its Origin, Characteristics, and Insights into Potential Drugs and Its Mechanisms. *Pathogens* **9**, 331 (2020).
19. Lu, X. et al. US CDC Real-Time Reverse Transcription PCR Panel for Detection of Severe Acute Respiratory Syndrome Coronavirus 2. *Emerg Infect Dis* **26**, 1654-1665 (2020).

Quiz for DNA simulation by NUPACK

1. What are the three factors affecting DNA hybridization stability?

2. What is the website for accessing NUPACK? Did you register an account with it?

3. Use **IC50** (https://www.aatbio.com/tools/ic50-calculator)to fit the Tm50 for dsDNA using the data set below:

Temperature	Fraction of unpaired
25	0.083582479
29	0.095704074
33	0.115209652
37	0.136696167
41	0.171084897
45	0.235084209
49	0.359035691
53	0.564163737
57	0.787051269
61	0.914698846
65	0.957051717
69	0.968961567
73	0.972632587
77	0.973967669
81	0.974538521
85	0.974817032

4. Use First-order derivative analysis to estimate Tm50 for the data set above.

Simulation of DNA hybridizations by NUPACK

NUPACK Registration (Free now)

- https://www.nupack.org/subscribe

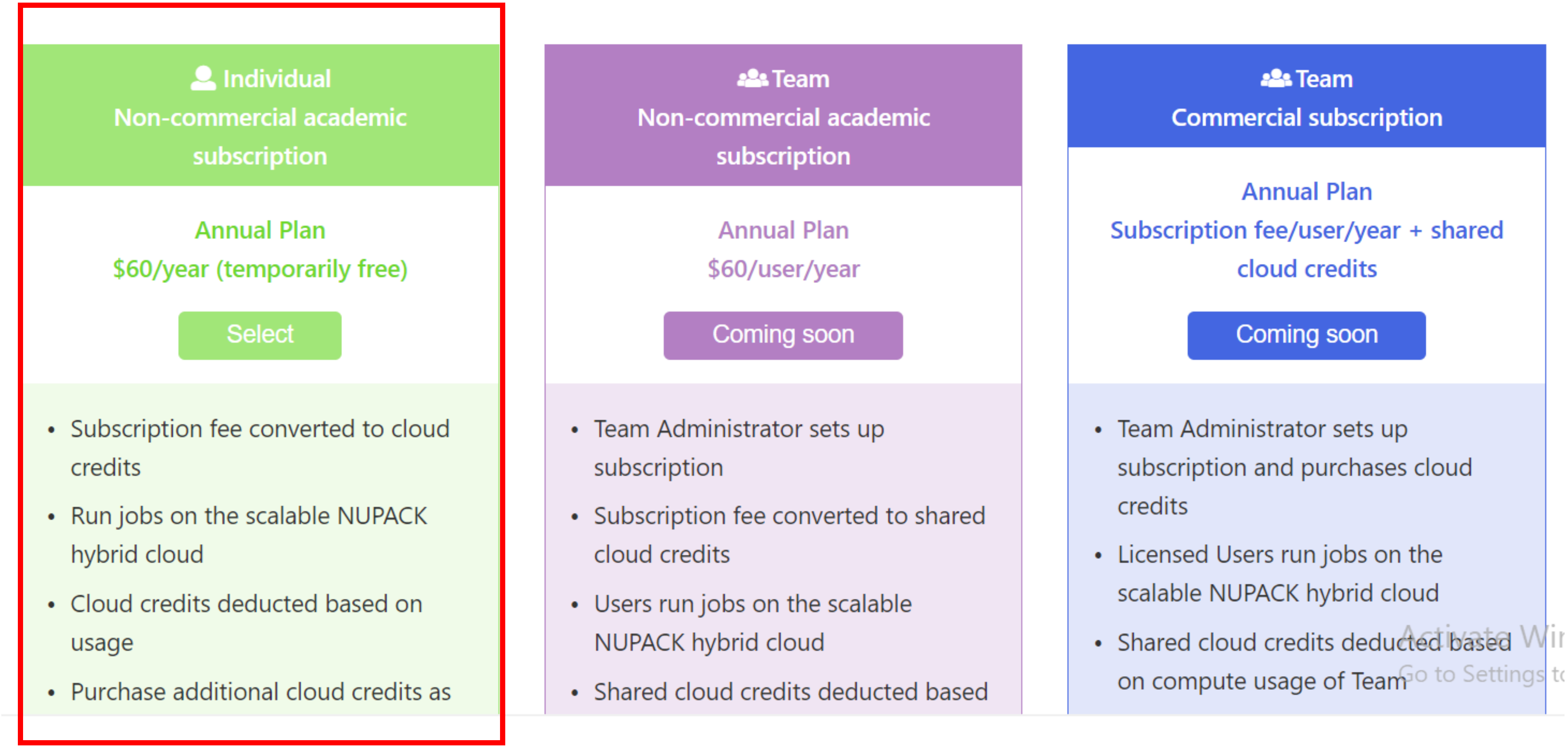

Register your use name on NUPACK Cloud for use it

DNA hybridization energy calculation

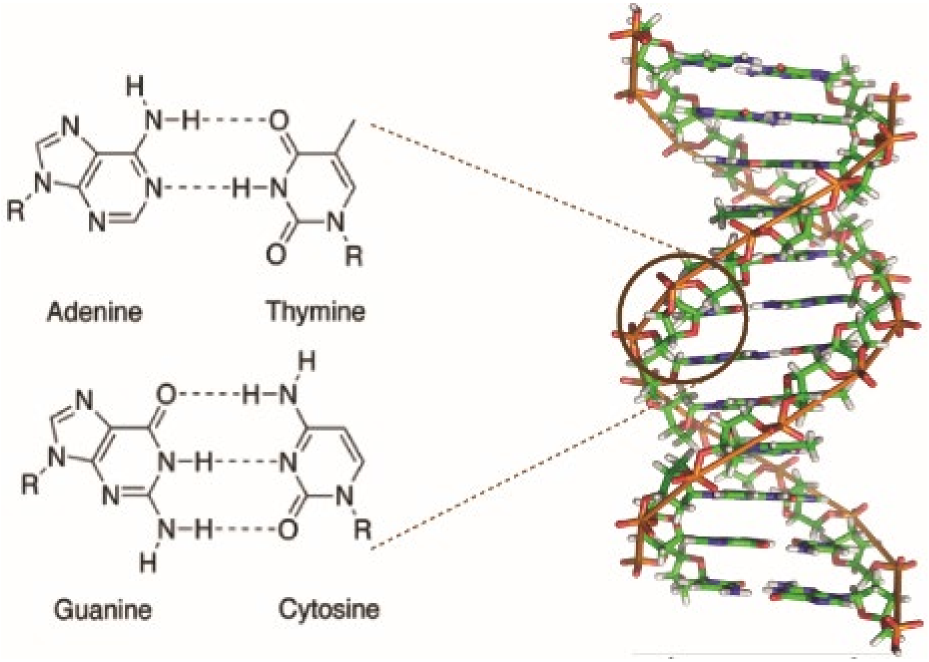

ssDNA: single-stranded

dsDNA: double-stranded

- dsDNA is stabilized by base pairing
- Hybridization energy follows a thermodynamic reaction:

$$\Delta G(total) = \sum \Delta G\ (individual\ base\ pairs) + \sum \pi - \pi\ stacking - \sum charge\ repulsion$$

- Melting temperature is an important parameter for DNA hybridization stability

DNA thermal melting (or thermal denaturation): The dsDNA complexes may be dissociated by thermal denaturation, which is referred to as thermal melting.

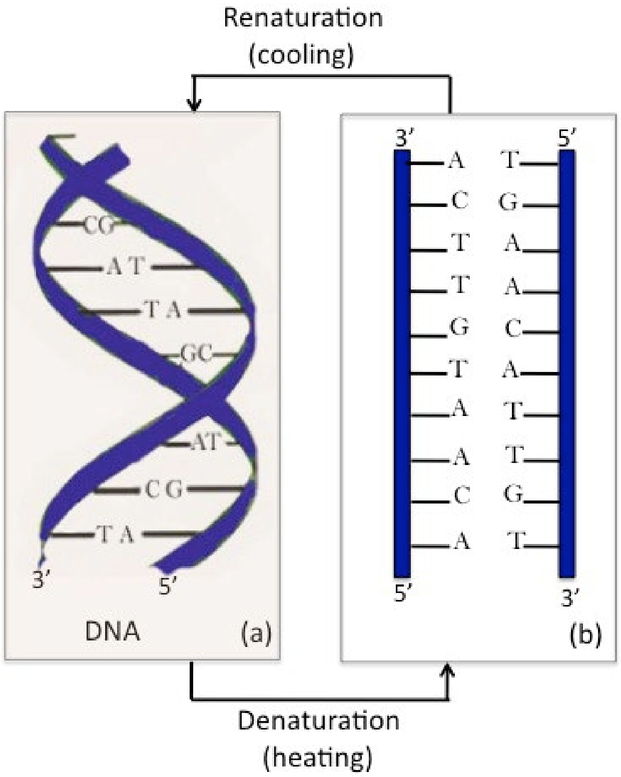

- Melting temperature (Tm): the melting temperature of DNA refers to the temperature at which 50% of DNA in a sample has denatured from double-stranded DNA (dsDNA) to single-stranded DNA (ssDNA). Sensitive measurement of the melting curve of a sample of DNA can be used to detect single nucleotide differences between two DNA samples.

- Other melting methods: Chemical reagents can also disrupt dsDNA, such as urea, SDS, detergent … It is called chemical denaturation.

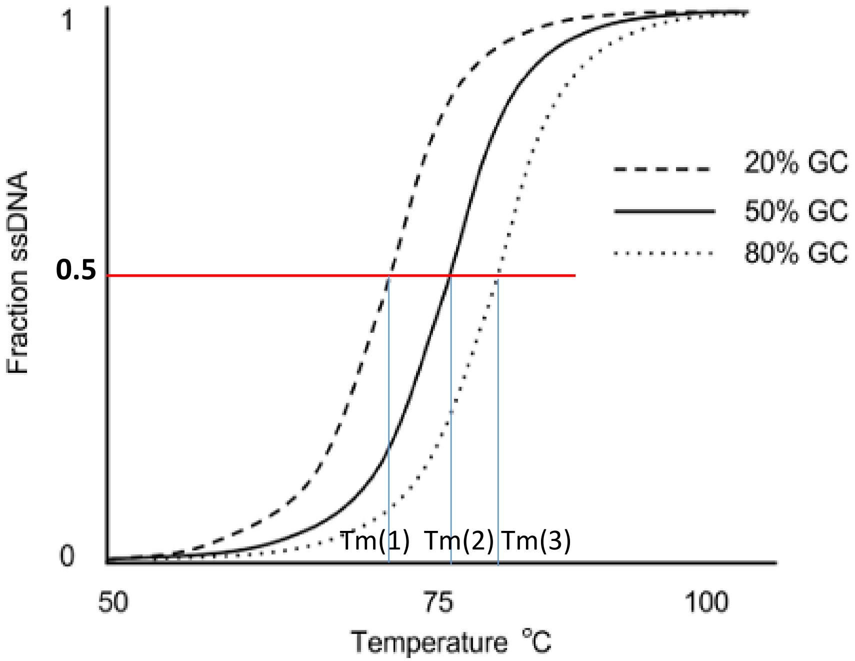

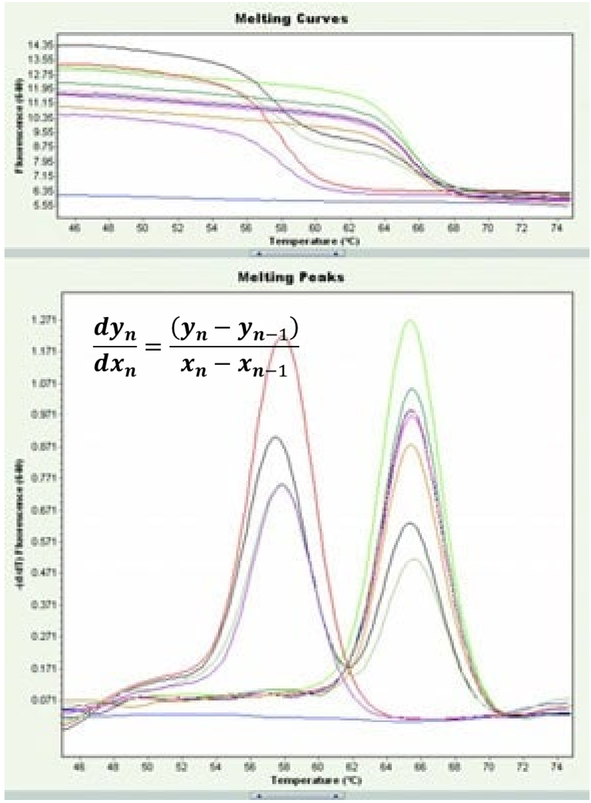

Melting Temperature (Tm) for dsDNA complexes with different GC%. Note that a dsDNA with **high GC%** shows a higher melting temperature due to the increased thermal stability. In addition, **magnesium ions** also play a key role in stabilizing dsDNA by shielding the negative charges of the phosphate backbone.

Tm, practically, is calculated by slope derivative analysis, which means that Tm is the temperature at the peak slope value. Generally, the first-order derivative analysis can be used to produce the slope curve and identify the Tm value

Lab Goal:

- **Learn NUPACK to simulate DNA hybridizations;**
- **Learn the fitting of DNA melting temperature;**
- **Understand how DNA melting temperature is impacted by GC%, length, and Mg2+ conc.**

DNA computation and simulation with NUPACK

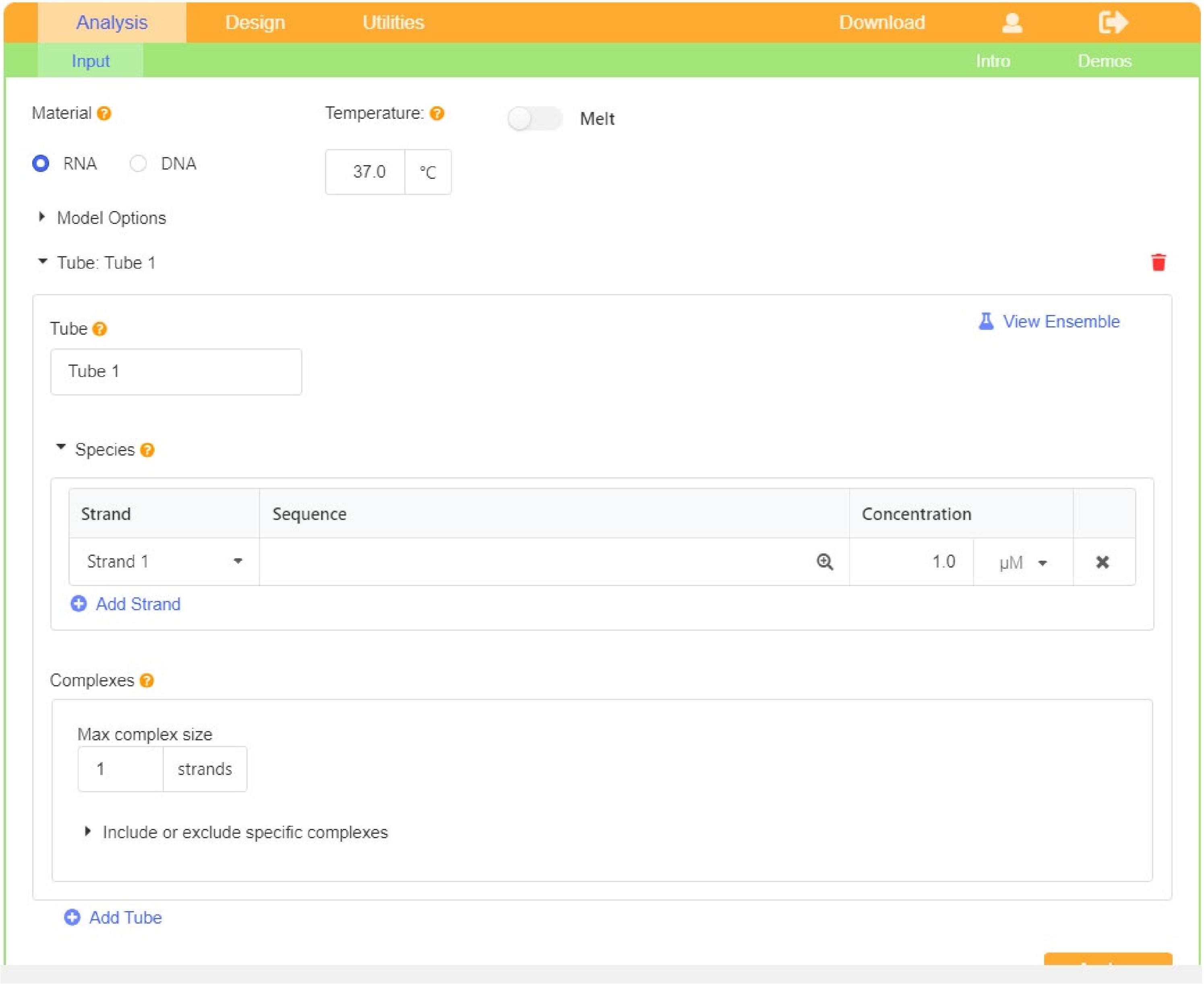

• Set Parameters for NUPACK

- Can analyze DNA and RNA;
- Set temperature and Melting analysis
- Run user-defined sequences and concentration
- In the “More Option”: Set Salt concentrations (Na+ and Mg2+)

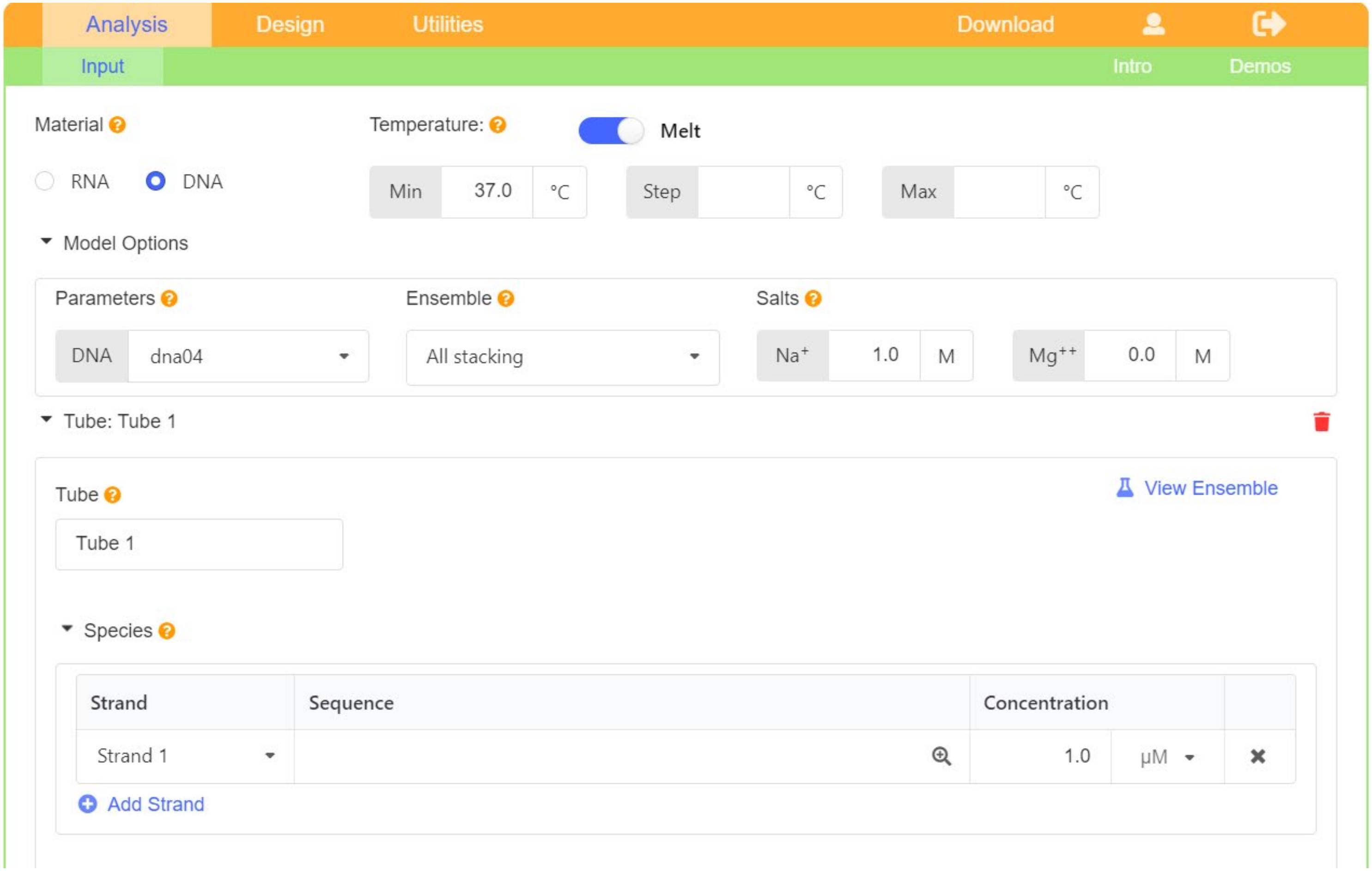

Task 1 Thermal Denaturation and Melting Temperature Fitting

- **Set Melting Temp analysis for poly (A-T) 20: Choose “DNA” and “Melt”**
- Set Min 25 C and Max 85 C, step 2 C
- Set “dna04” and “All stacking”; set Na+ to 0.137 M, Mg++ to 0 M
- Set the name of “Tube” as: poly (A-T)20
- Set two strands of poly A20 and polyT20. You can use “Add strand” to add the second strand. Conc. = 1 uM

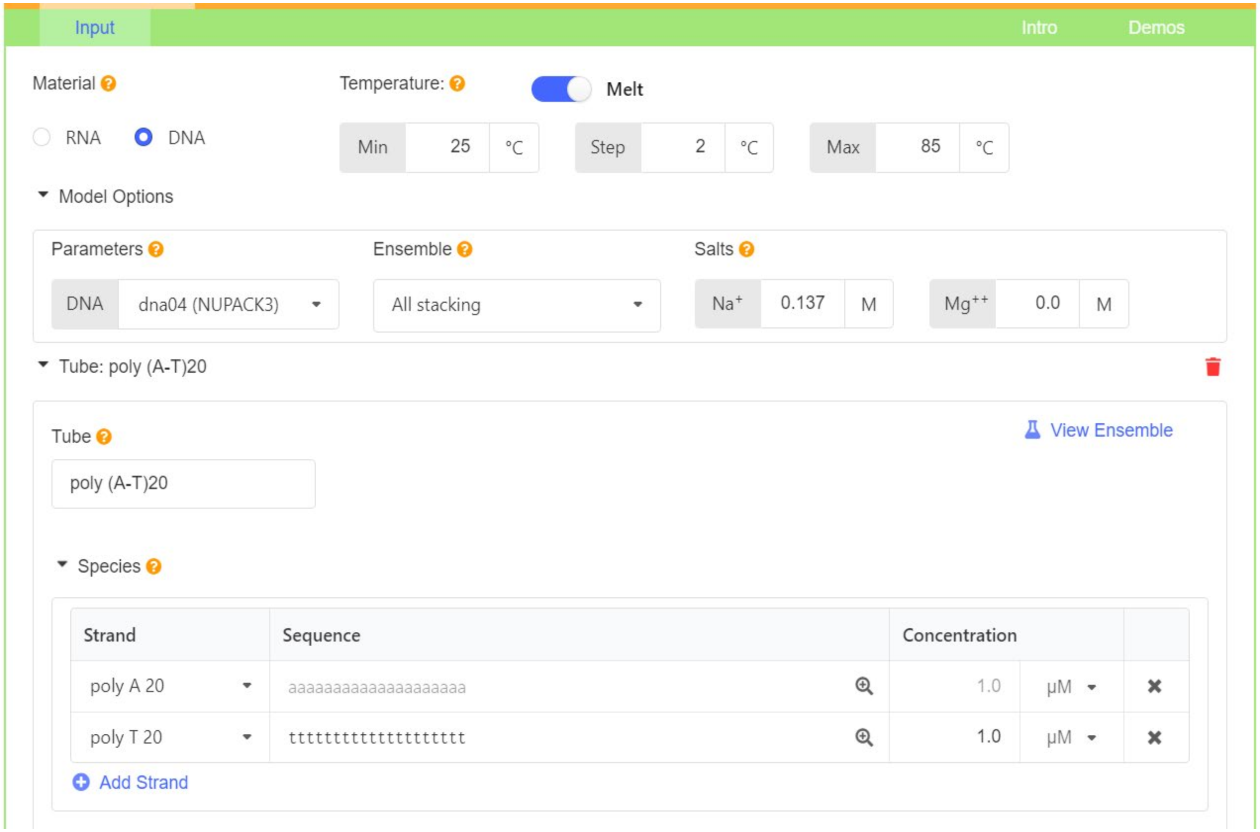

Task 1 Thermal Denaturation and Melting Temperature Fitting

- Set “Max Complex Size” to 2 strands for dsDNA
- Click “Analyze”

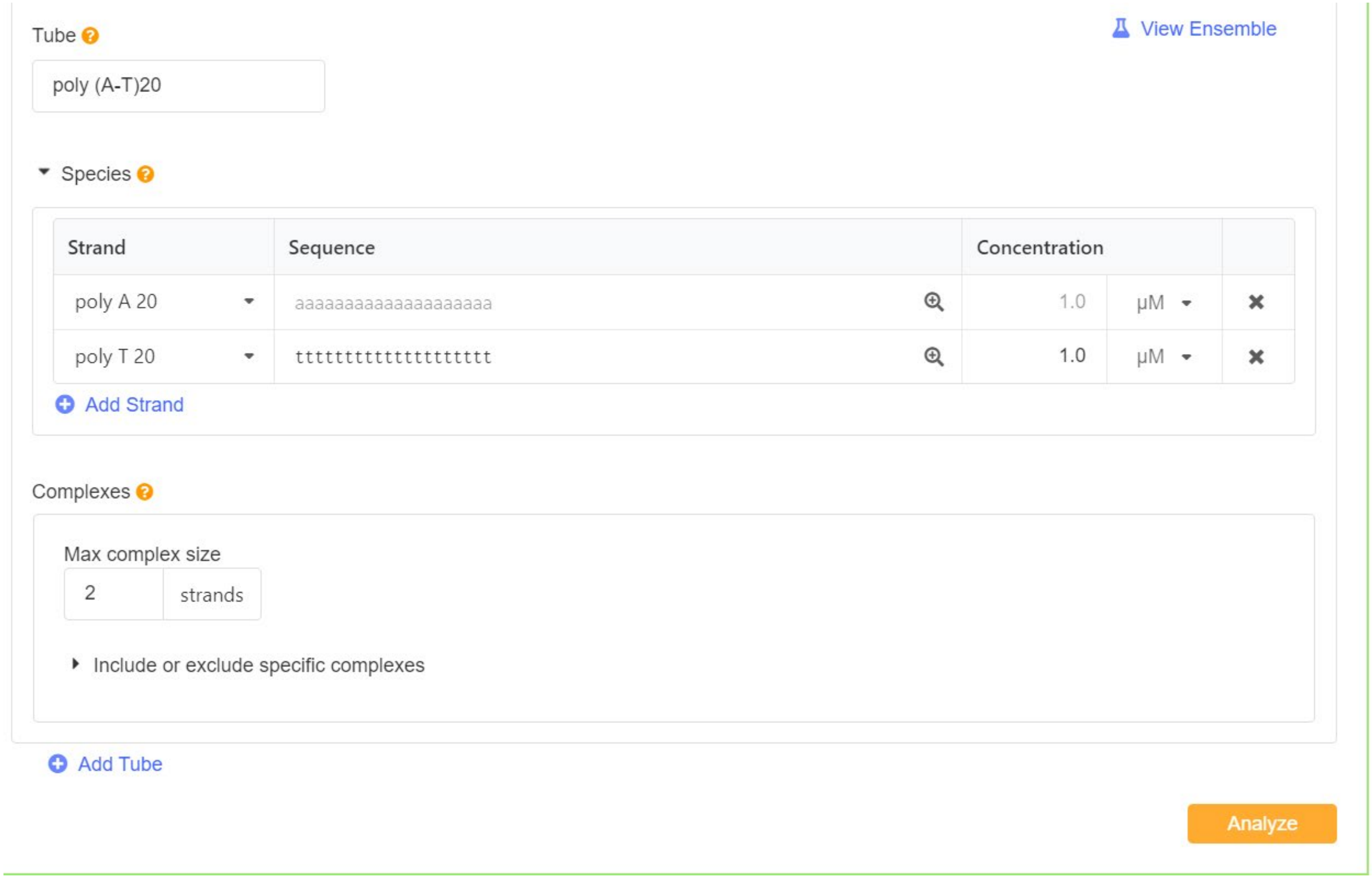

Task 1 Thermal Denaturation and Melting Temperature Fitting

- Click results from analysis view melting graph.
- You can drag the temperature bar to view the hybridization changes at a specific temperature

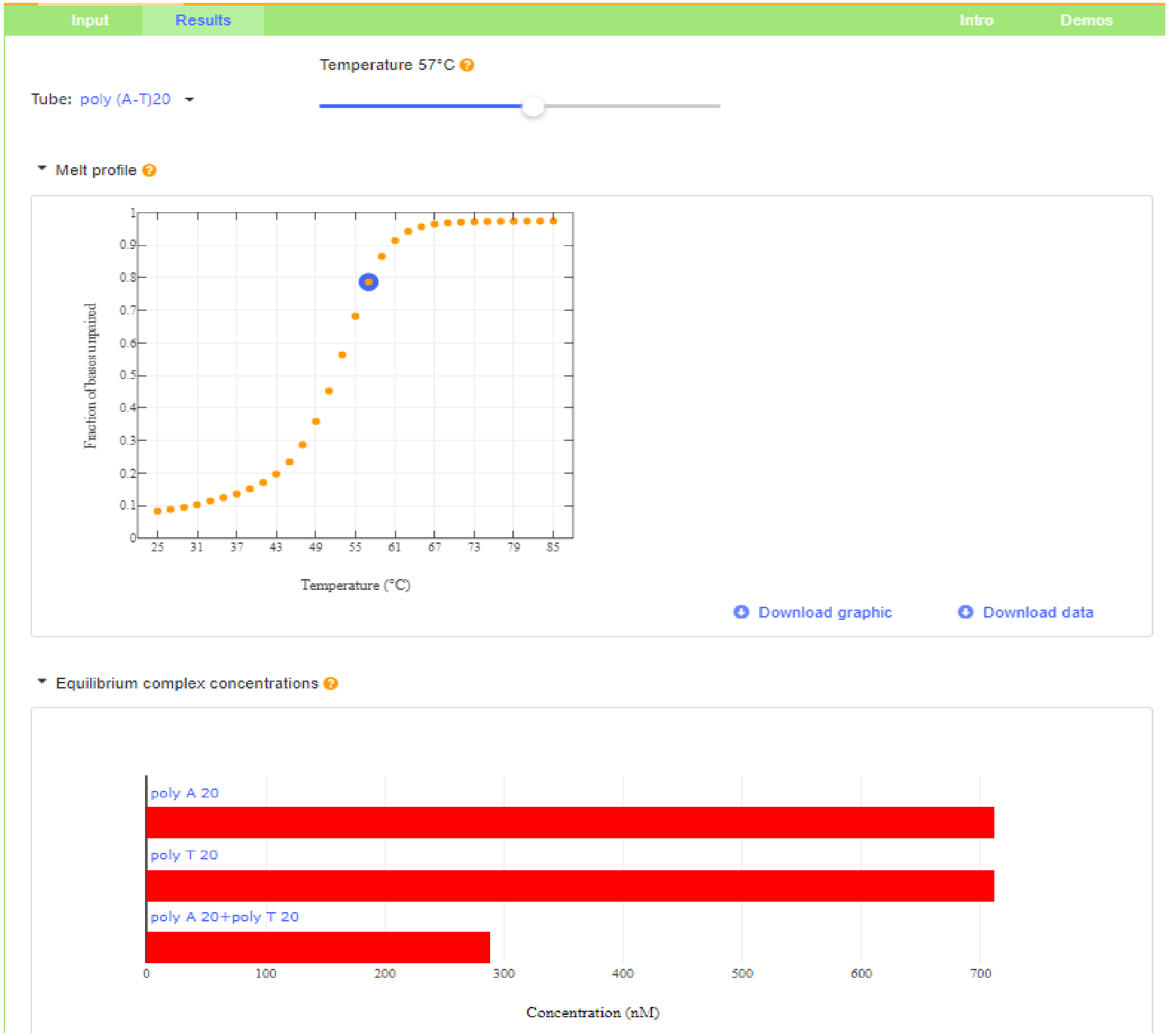

Task 1 Thermal Denaturation and Melting Temperature Fitting

- Download data from NUPACK simulation
- Click "Download" to save Excel's simulated DNA hybridization data.

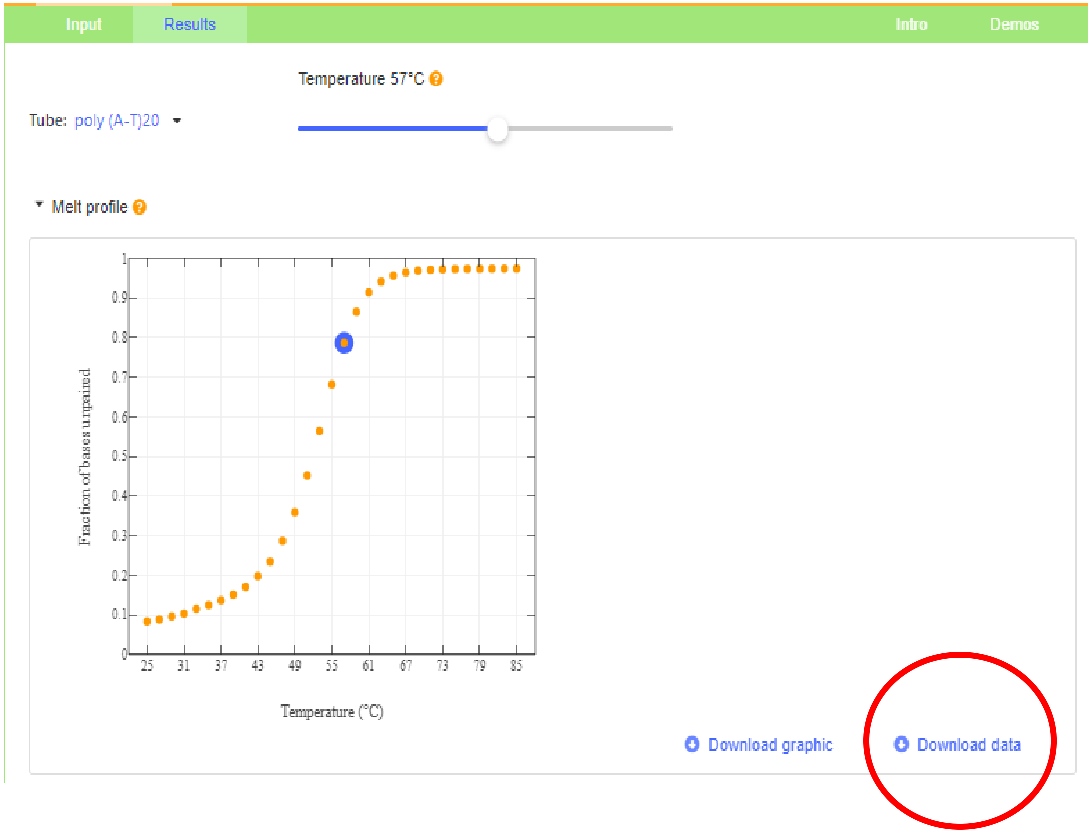

A	B	C
Temperature(C)	Fraction of bases un[illegible]	
25	0.083582	
27	0.088888	
29	0.095704	
31	0.103196	
33	0.11521	
35	0.124953	
37	0.136696	
39	0.151554	
41	0.171085	
43	0.197727	
45	0.235084	
47	0.287373	
49	0.359036	
51	0.452491	
53	0.564164	
55	0.681804	
57	0.787051	
59	0.865391	
61	0.914699	

• Fitting of "Melt Temp" from downloaded data

- Tm fitting with IC50 online tool: https://www.aatbio.com/tools/ic50-calculator
- Copy and paste melting data into the blank box of Data Entry, then click Process Data. Calculate IC50 is displayed as the Melt Temp of dsDNA.

Data Entry

Load Data | Save Data | Import from File

Dataset 1 | +

```
25 0.083
27 0.088
29 0.096
31 0.103
33 0.115
35 0.125
37 0.137
```

IC_{50} Regression Results [Dataset 1]

Parameter	Value
IC_{50}	52.6517
Equations	
Equation	$Y = 0.114 + \frac{1.0149 - 0.114}{1 + \left(\frac{X}{52.6517}\right)^{-13.1019}}$
Equation Form	$Y = Min + \frac{Max - Min}{1 + \left(\frac{X}{IC50}\right)^{\text{Hill coefficient}}}$

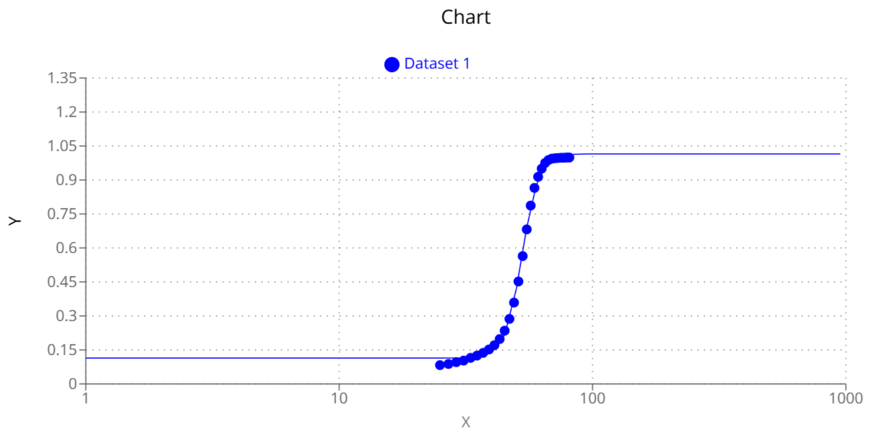

Task 1 Thermal Denaturation and Melting Temperature Fitting

All NUPACK experiments are set at 0.137 M Na^+, temperature ranges from 20 ºC to 80 ºC.

1. NUPACK-generated thermal denaturation graph for poly$(A\text{-}T)_{20}$ and poly $(A\text{-}T)_{40}$
2. NUPACK-generated thermal denaturation graph for poly$(A\text{-}T)_{20}$ and poly $(C\text{-}G)_{20}$
3. NUPACK-generated thermal denaturation graph for poly$(A\text{-}T)_{20}$ at 0 Mg^{2+} and poly$(A\text{-}T)_{20}$ at 0.01 M Mg^{2+}
4. Melting temperature fitting by at least two methods of "Boltzmann Sigmoidal Kinetics" (or IC50) and "the first order derivative analysis."
5. Summary of the melting temperatures for DNA hybridizations above. Can you conclude what factors affect the melting temperature of dsDNA hybridization?

Task 2. Thermal Denaturation for Single Nucleotide Polymorphism

A single nucleotide polymorphism, or SNP (pronounced "snip"), is a variation at a single position in an individual's DNA sequence. For example, at a specific base position in the human genome, the G nucleotide may appear in most individuals. Still, in a minority of individuals, the position is occupied by an A. This means there is an SNP at this specific position, and the two possible nucleotide variations – G or A – are said to be the alleles for this specific position. Although a particular SNP may not cause a disorder, some SNPs are associated with certain diseases.

SNP may or may not cause an amino acid mutation.

A single-nucleotide variant (SNV) is a variation in a single nucleotide. SNVs differ from SNPs in that an SNV can be somatic[9] and caused by cancer,[10] but an SNP has to segregate in a species' population of organisms. SNVs also commonly arise in molecular diagnostics, such as designing PCR primers to detect viruses, in which the viral RNA or DNA sample may contain SNVs.

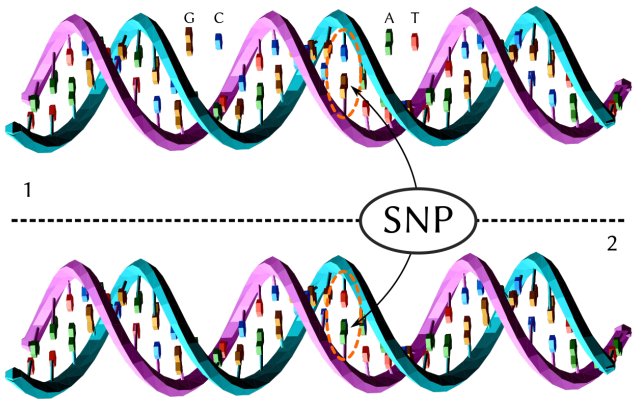

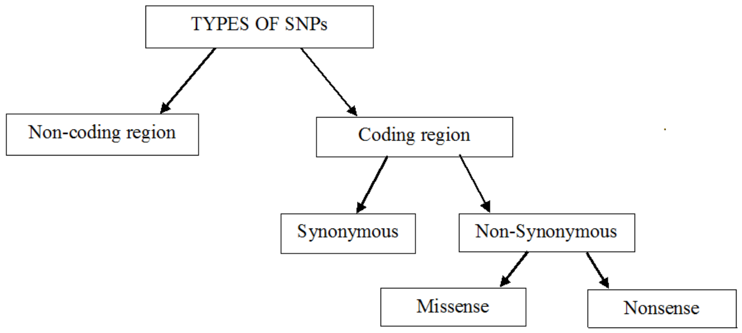

https://en.wikipedia.org/wiki/Single-nucleotide_polymorphism#/media/File:Dna-SNP.svg

SNP/SNV detection by PCR with melting temperature difference, **first-order derivative analysis**

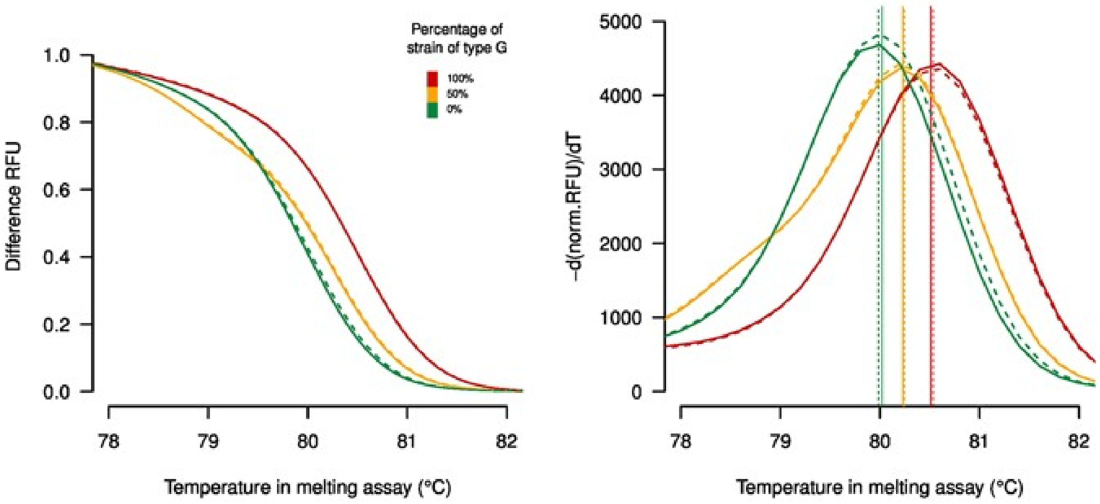

Derivative Analysis with Excel

$$\frac{dy_n}{dx_n} = \frac{(y_n - y_{n-1})}{x_n - x_{n-1}}$$

Temperature	A260	Derivative
25	1.12084	
30	1.12119	=(B3-B2)/(A3-A2)
35	1.12882	0.001526403
40	1.13616	0.001466918
45	1.17456	0.00768013
50	1.2033	0.00574801
55	1.21659	0.002658391
60	1.21771	0.000224924
65	1.22205	0.00086658
70	1.22659	0.000908089
75	1.23146	0.000974798
80	1.23686	0.001080463

Tm ~ 46 C

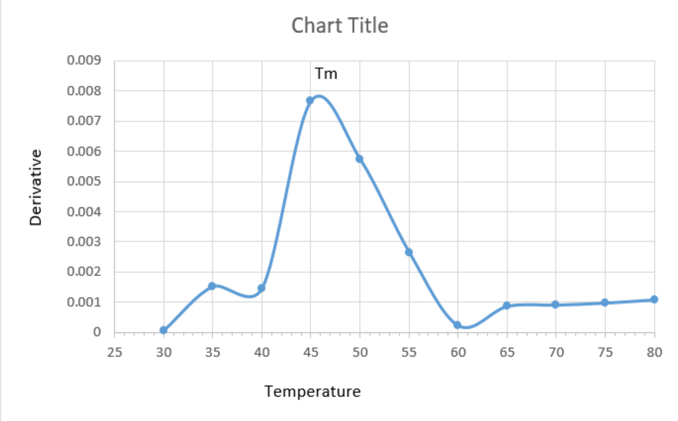

Tm is the temperature with the highest derivative value.

Example: CYP1A2 SNP

rs762551, also known as -164A>C or -163C>A, is a SNP encoding the CYP1A2*1F allele of the CYP1A2 gene. The CYP1A2 gene encodes a member of the cytochrome p450 family of proteins, which metabolize nutrients and drugs. One well-known substrate of CYP1A2 is caffeine; individuals who carry one or more CYP1A2*1C alleles are "slow" caffeine metabolizers, whereas carriers of the variant CYP1A2*1F are "fast" caffeine metabolizers.

Primer	Sequences
TaqMan probe – TET	5′-CTC TGT GGG C**C**C AGG ACG CAT-3′
TaqMan probe – FAM	5′-TC TGT GGG CA**C** AGG ACG CAT GG-3′
TaqMan Forward primer	5′-TTT CCA GCT CTC AGA TTC TGT GAT-3′
TaqMan Reverse primer	5′-GGA TAC CAG AAA GAC TAA GCT CCA TC-3′
Forward primer*	5′-TTC CCC ATT TTG GAG TGG TC-3′
Reverse primer*	5′-CCG AGA AGG GAA CAG ACT GG-3′

Nordmark et. al. 2002

Home › Search Tool › Search Results › C___8881221_40

**SNP ID:**	rs762551
**Gene**	▾CYP1A2
**Gene Name**	cytochrome P450 family 1 subfamily A member 2
**Set Membership:**	> HapMap > DME > Validated > Inventoried
**Chromosome Location:**	-
**Polymorphism:**	C/A, Transversion Substitution
**Context Sequence [VIC/FAM]:**	TGCTCAAAGGGTGAGCTCTGTGGGC[C/A]CAGGACGCATGGTA GATGGAGCTTA

Task 2: Use Nupack to predict thermal melting graphs and Tm at 0.137 M Na+ and 0 Mg++

1) Anti-TET + TET seq (1 uM) and anti-TET + FAM seq (1 uM)

	Sequence	Tm (C)
Target TET	CTCTGTGGGCCCAGGACGCAT	
Target FAM	CTCTGTGGGCACAGGACGCAT	
Anti-TET	ATGCGTCCTGGGCCCACAGAG	

2) Based on the Tm difference, suggest **a temperature** that produces the most significant difference for hybridizations between TET/anti-TET and FAM/anti-TET

	Sequence	Test Temperature (C)	Hybridization yield (%)
Target TET	CTCTGTGGGCCCAG GACGCAT		
Target FAM	CTCTGTGGGCACA GGACGCAT		

• **Melting Temp by first-order derivative analysis**

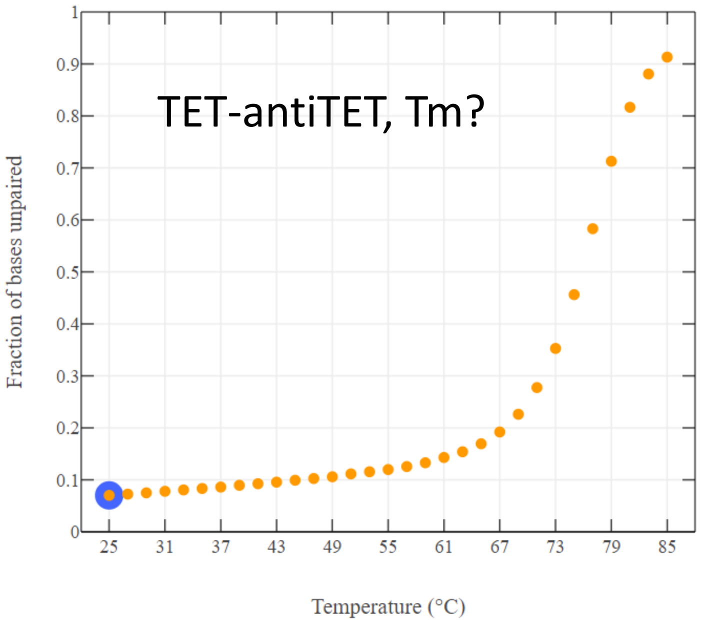

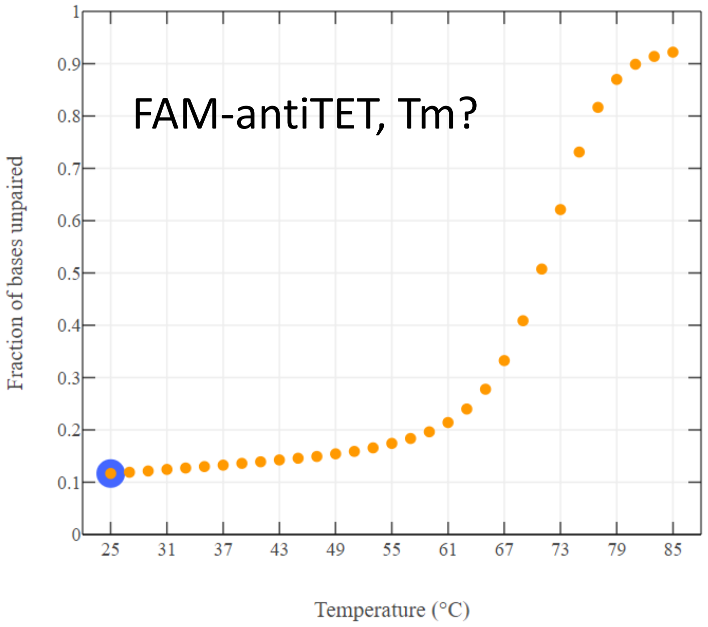

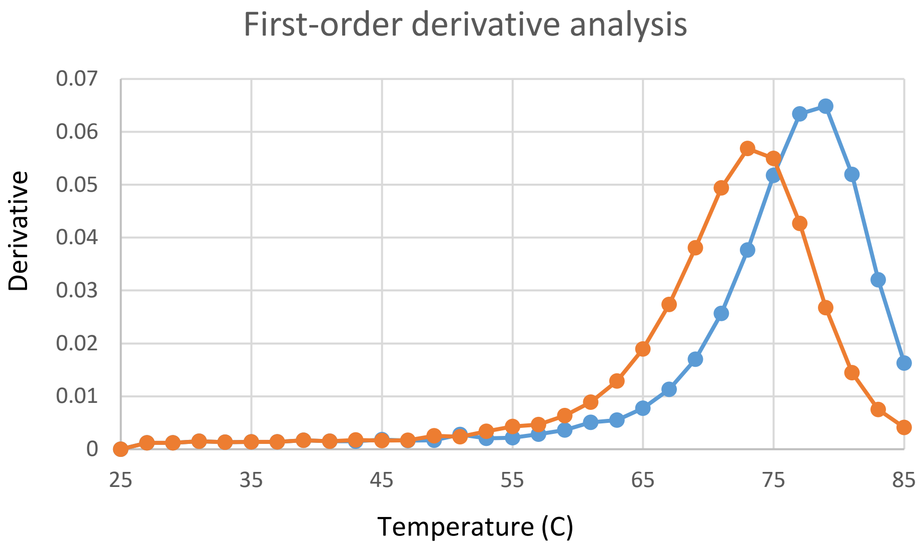

Hybridization Yield%

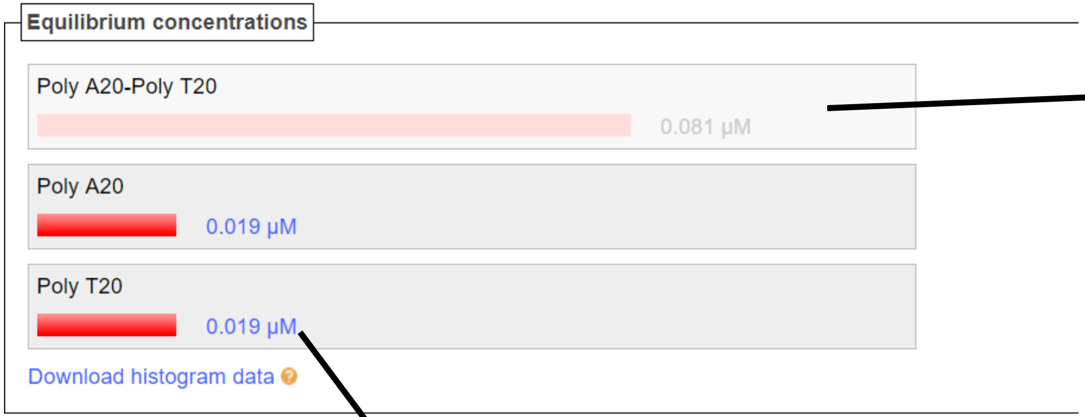

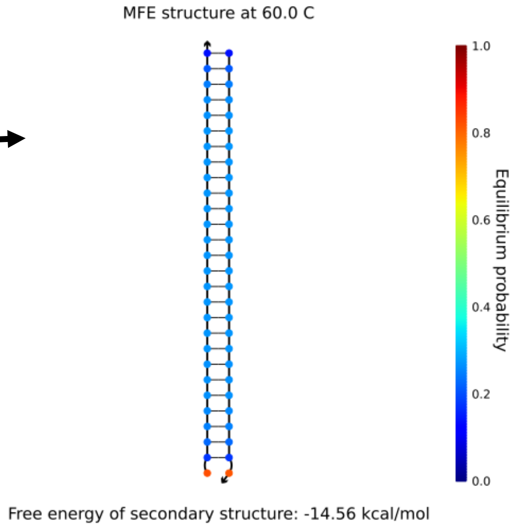

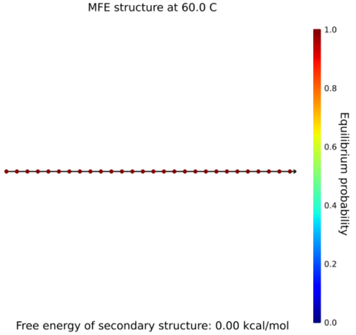

The color means the probability of the base in the predicted structure: Red, high probability; blue, low probability

Nupack can predict the equilibrium conc. of hybridizations. You can calculate the product yield:

e.g.

[Target dsDNA]/[total ssDNA input] = 0.081/0.1 = 0.81=81%

Task 3. Simulation of the secondary folding structures of ssDNA amplicons

the folding structure of SARS-COV-2 (cause COVID-19) amplicons for PCR diagnosis

N1 :
GATAATGGACCCCAAAATCAGCGAAATGCACCCCGCATTACGTTTGGTGGACCCTCAGATTCAACTGG CAGTAACCAGAAT

N2:
ACTAATCAGACAAGGAACTGATTACAAACATTGGCCGCAAATTGCACAATTTGCCCCCAGCGCTTCAG CGTTCTTCGGAATGTCGCGC

N3:
AGACGGCATCATATGGGTTGCAACTGAGGGAGCCTTGAATACACCAAAAGATCACATTGGCACCCGC AATCCTGCTAACAATGCTGCAATCGTGCTACAACTTCCTCAAGGAACAACA

The color means the probability of the base in the predicted structure: Red, high probability; blue, low probability.

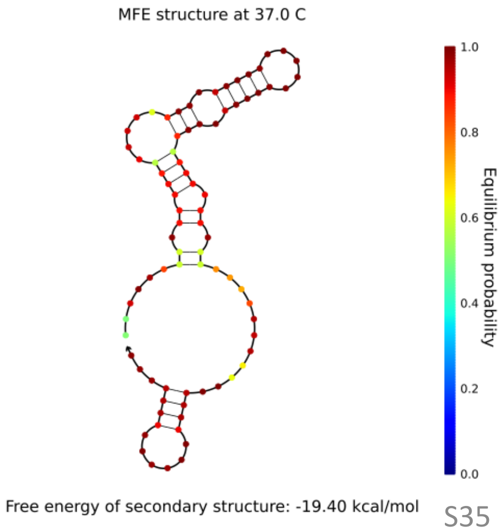

- **Calculation mode for RNA**
- **Under RNA mode, you cannot choose salt concentration**
- **Set Temp to 37 C, 1 strand**

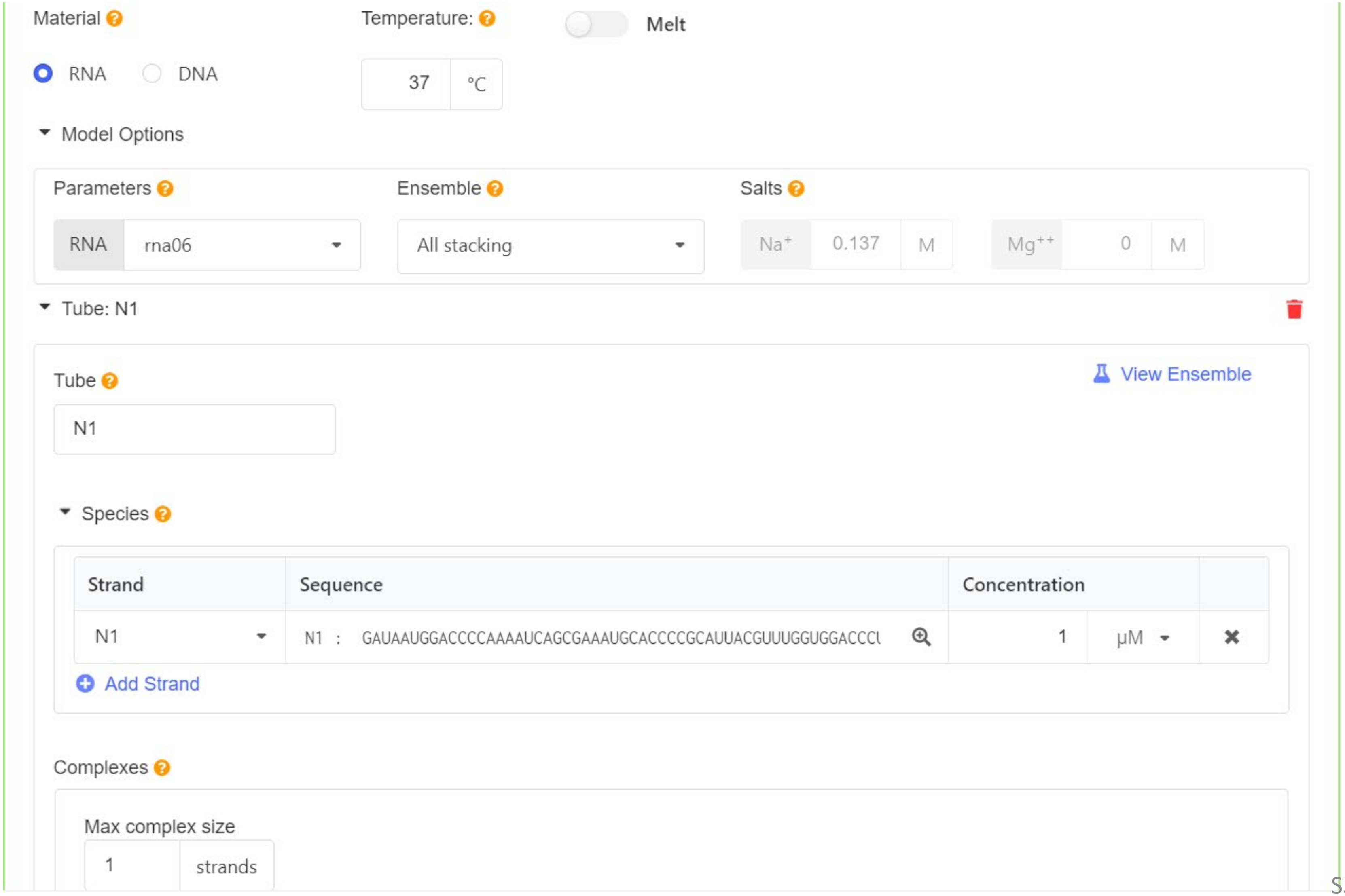

MFE proxy structure at 37°C

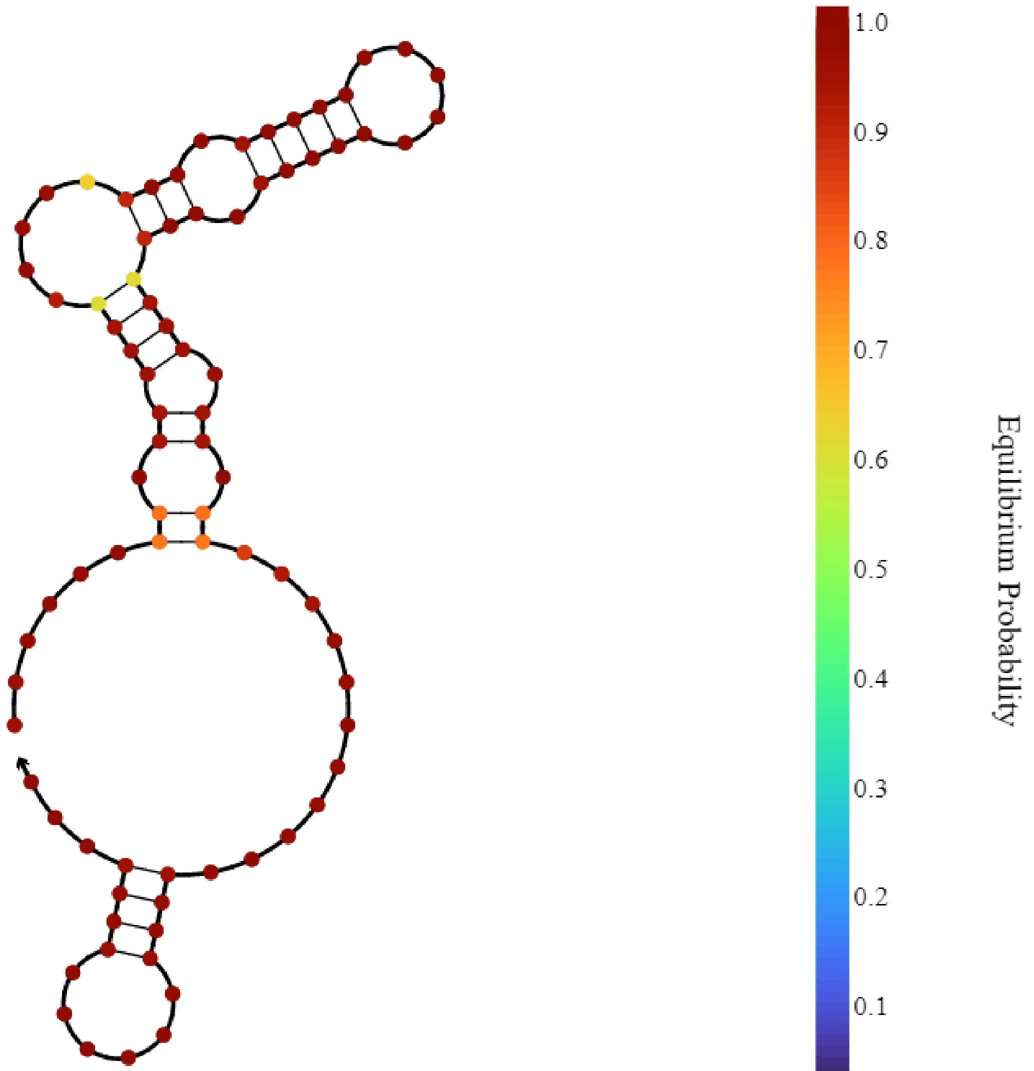

Free energy of the secondary structure: -15.42 kcal/mol

A data report is required as follows:

Task 1. Thermal Denaturation and Melting Temperature Fitting

All NUPACK experiments are set at 0.137 M Na^+, temperature ranges from 20 ^oC to 80 ^oC.

1. NUPACK-generated thermal denaturation graph for poly$(A\text{-}T)_{20}$ and poly $(A\text{-}T)_{40}$
2. NUPACK-generated thermal denaturation graph for poly$(A\text{-}T)_{20}$ and poly $(C\text{-}G)_{20}$
3. NUPACK-generated thermal denaturation graph for poly$(A\text{-}T)_{20}$ at 0 Mg^{2+} and poly$(A\text{-}T)_{20}$ at 0.01 M Mg^{2+}
4. Melting temperature fitting by at least two methods of "Boltzmann Sigmoidal Kinetics" (or IC50) and "the first order derivative analysis."
5. Summary of the melting temperatures for DNA hybridizations above. Can you conclude what factors affect the melting temperature of dsDNA hybridization?

Task 2. Thermal Denaturation for Single Nucleotide Polymorphism

All NUPACK experiments are set at 0.137 M Na^+

2.1. NUPACK-generated thermal denaturation graphs for TET hybridizing with Target and FAM hybridizing with Target.

2.2. Melting temperature fitting by "IC50" and "the first order derivative analysis."

2.3. Pick a temperature based on the melting temperature fitting with a significant difference in hybridization yield between TET-Target and FAM-Target.

2.4. Can you conclude how the single-nucleotide mismatch affects the melting temperature of the DNA hybridization?

Task 3. Simulation of the secondary folding structures of ssDNA amplicons

3.1. Simulation of the secondary folding structures of N1, N2, and N3 ssDNA amplicons at 25 ^{o}C and ΔG of structures.

3.2. Simulation of the secondary folding structures of N1, N2, and N3 ssDNA amplicons at 60 ^{o}C and ΔG of structures.

3.3. Simulation of the secondary folding structures of N1, N2, and N3 ssDNA amplicons at 90 ^{o}C and ΔG of structures.

3.4. Can you conclude how the secondary folding structures of ssDNA change depending on the temperature and their structural ΔG?
